# Hierarchical Temporal Processing in the Primate Thalamocortical System: Insights from Nonlinguistic Structured Stimuli

**DOI:** 10.34133/research.0960

**Published:** 2025-11-06

**Authors:** Hangting Ye, Peirun Song, Haoxuan Xu, Qiuyu Li, Yanxin Chen, Yuying Zhai, Cheng Chang, Xuehui Bao, Hisashi Tanigawa, Zhiyi Tu, Pei Chen, Tingting Zhang, Lingling Zhang, Xuan Zhao, Wanshun Wen, David Pérez-González, Manuel S. Malmierca, Xiongjie Yu

**Affiliations:** ^1^ Department of Anesthesiology, Shanghai Tenth People’s Hospital, Tongji University School of Medicine, Shanghai, China.; ^2^ Department of Anesthesia, Women’s Hospital, Zhejiang University School of Medicine, Hangzhou, Zhejiang, China.; ^3^ Oujiang Laboratory (Zhejiang Lab for Regenerative Medicine, Vision and Brain Health), Wenzhou, Zhejiang, China.; ^4^ Zhejiang Key Laboratory of Precision Diagnosis and Therapy for Major Gynecological Diseases, Women’s Hospital, Zhejiang University School of Medicine, Hangzhou, Zhejiang, China.; ^5^ College of Biomedical Engineering and Instrument Science, Zhejiang University, Hangzhou, Zhejiang, China.; ^6^ Key Laboratory for Biomedical Engineering of Ministry of Education, Zhejiang University, Hangzhou, Zhejiang, China.; ^7^ Center for Rehabilitation Medicine, Rehabilitation and Sports Medicine Research Institute of Zhejiang Province, Department of Rehabilitation Medicine, Zhejiang Provincial People’s Hospital, Affiliated People’s Hospital, Hangzhou Medical College, Hangzhou, Zhejiang, China.; ^8^ Cognitive and Auditory Neuroscience Laboratory (Lab 1), Institute of Neuroscience of Castilla y León (INCYL), University of Salamanca, Salamanca, Spain.; ^9^ Institute for Biomedical Research of Salamanca (IBSAL), Salamanca, Spain.; ^10^ Department of Basic Psychology, Psychobiology and Methodology of Behavioral Sciences, Faculty of Psychology, University of Salamanca, Salamanca, Spain.; ^11^ Department of Cell Biology and Pathology, Faculty of Medicine, University of Salamanca, Salamanca, Spain.

## Abstract

The ability of the brain to process auditory information across multiple temporal scales is crucial for perception. This study introduces an innovative experimental approach using nonlinguistic structured stimuli composed of click trains that alternate at different rates to investigate auditory processing across 3 distinct temporal scales: individual clicks (tens of milliseconds), click trains forming auditory objects (hundreds of milliseconds), and higher-order click trains in neuronal novelty detection (seconds) in both rhesus monkeys and humans. Electrocorticography recordings in the auditory cortex of rhesus monkeys unveil the primate brain’s remarkable ability to process intricate auditory temporal patterns at timescales of tens and hundreds of milliseconds. Furthermore, extracellular recordings in monkeys demonstrate pronounced responsiveness to deviant click trains at longer temporal scales in the primary auditory cortex (A1), accompanied by synchronization with both individual clicks and click trains. By contrast, neurons in the auditory thalamus prefer individual clicks only. Notably, neurons in A1 exhibit the ability to synchronize with individual clicks while simultaneously integrating these clicks into cohesive train objects, a phenomenon we term “temporal integration during synchronization”. Additional human electroencephalography recordings complement and support these findings, highlighting the paradigm’s potential for clinical applications. Our research offers novel insights into the neural mechanisms underpinning auditory information processing across various temporal scales at the neuronal level.

## Introduction

Sound inherently carries temporal information, making time a fundamental dimension in auditory perception. Within auditory processing, the critical component of temporal processing operates across various timescales [1]. Notably, previous studies have unveiled the extraordinary capacity of the human auditory system to track temporal structures across diverse scales. For instance, in the realm of spoken language, the brain exhibits discernible neural responses to individual syllables, phrases, and sentences, underscoring its finely tuned sensitivity to nuanced temporal variations [2,3]. Moreover, various brain regions have demonstrated selectivity to words, sentences, and even paragraphs, suggesting a hierarchical organization in the processing of temporal information [4,5]. This perspective suggests that temporal processing could serve as a functional characteristic, providing a potential overarching principle for our understanding of the organization of the cerebral cortex [1]. However, it is important to note that most investigations of temporal processing across multiple scales have been conducted in humans, typically using spoken language and macroscopic recording techniques [2,4,5], which together limit direct access to underlying neuronal mechanisms. Therefore, animal models offer a powerful and practical approach for probing temporal processing in audition. To support such research, there is a critical need for stimuli that capture multiple temporal scales beyond those found in spoken language.

To create such stimuli, we will use click trains, as they offer an ideal avenue for investigating temporal processing within the auditory system. Click trains consist of identical pulses with varying temporal characteristics and have been extensively employed in auditory research [6–9]. However, they have not yet been exploited in the context of multiple temporal scale processing, where each click could be associated with a syllable that forms higher-order sound structures. Previous studies primarily focused on the encoding of individual clicks, examining temporal and rate encoding across different click rates [6,7]. Psychological studies have demonstrated that the click train can be perceived as a continuous pitch-like sound when the inter-click interval (ICI) is sufficiently short [10,11]. This indicates that a click train can be perceived as an auditory object (temporal pitch), and it is made of a long-time scale structure but composed of individual clicks with short-time scale characteristics. This transformation from discrete pulses to a unified percept supports the notion that click trains function as auditory objects, composed of rapid, fine-grained elements integrated into a coherent, longer-time scale structure. Such object-level encoding provides a substrate for predictive mechanisms operating on extended timescales, such as those engaged by oddball paradigms. These paradigms, often using pure tones, have been widely used to probe mismatch negativity (MMN) at the electroencephalography (EEG) level [12–14] and stimulus-specific adaptation (SSA) at the single-neuron level [15–25]—both thought to reflect predictive coding processes acting over longer time to generate mismatch signals. Despite the prevalence of clicks in basic auditory research, the potential for click trains to serve as structured pitch-like objects in this context has received limited attention. We propose that the individual clicks (fine timescale, first level), the formation of temporal pitch or click trains (intermediate timescale, second level), and higher-order click trains as a context-dependent, predictive process (long timescale, third level) together form a 3-tiered hierarchy of temporal processing—extending beyond traditional speech-based analogies of syllables, phrases, and sentences to reveal core organizational principles of auditory computation.

Importantly, deficits in temporal integration have been consistently reported in psychiatric conditions such as schizophrenia [26,27], where attenuated MMN responses reflect impaired predictive coding [28], as well as in autism spectrum disorders [29,30], attention deficit hyperactivity disorder [31], and Parkinson’s disease [32]. Given that oddball paradigms are already clinically employed to probe atypical mental conditions, a paradigm specifically designed to engage auditory processing across multiple temporal scales provides a mechanistically grounded extension with translational potential. Nevertheless, it remains essential to establish whether hierarchical temporal processing is robustly and consistently elicited in the human brain. Demonstrating this phenomenon in healthy and neurotypical populations represents a critical step toward assessing the generalizability of this framework in auditory neuroscience and evaluating its prospective clinical utility.

To address these questions, we developed a novel stimulus paradigm composed of click trains specifically designed to isolate and probe auditory processing at 3 distinct temporal scales within a unified experimental framework. Our results demonstrate that neurons in the primary auditory cortex (A1) are remarkably well suited to deciphering these temporal structures across multiple scales, ranging from the swift tens of milliseconds in click intervals, through the longer hundreds of milliseconds in click trains to the more extended periods of seconds in higher-order click trains. Conversely, the medial geniculate body (MGB) in the auditory thalamus primarily focuses on the first-level signals. Through parallel experiments in humans and macaques, we aimed to elucidate both the conserved and divergent features of temporal integration in the auditory system [33], and to provide a framework for translating mechanistic insights from animal models to human neuroscience and potential clinical applications. In this study, we aim to shed light on the foundational mechanisms by which the auditory brain decodes complex temporal structures, broadening investigation from human to animal models, from language to nonlanguage sounds, and from macroscopic to microscopic perspectives, to gain a comprehensive understanding of auditory temporal processing.

## Results

### Simultaneous tracking of individual clicks and click trains

An interesting aspect of regular click-train perception is that they are perceived as having pitch-like qualities when the repetition rate exceeds approximately 30 Hz [10,11]. This observation suggests that click trains contain 2 distinct pieces of information: individual click and the perception of the entire click train as a pitch-like object. These 2 components correspond to different temporal scales.

**Fig. 1.**
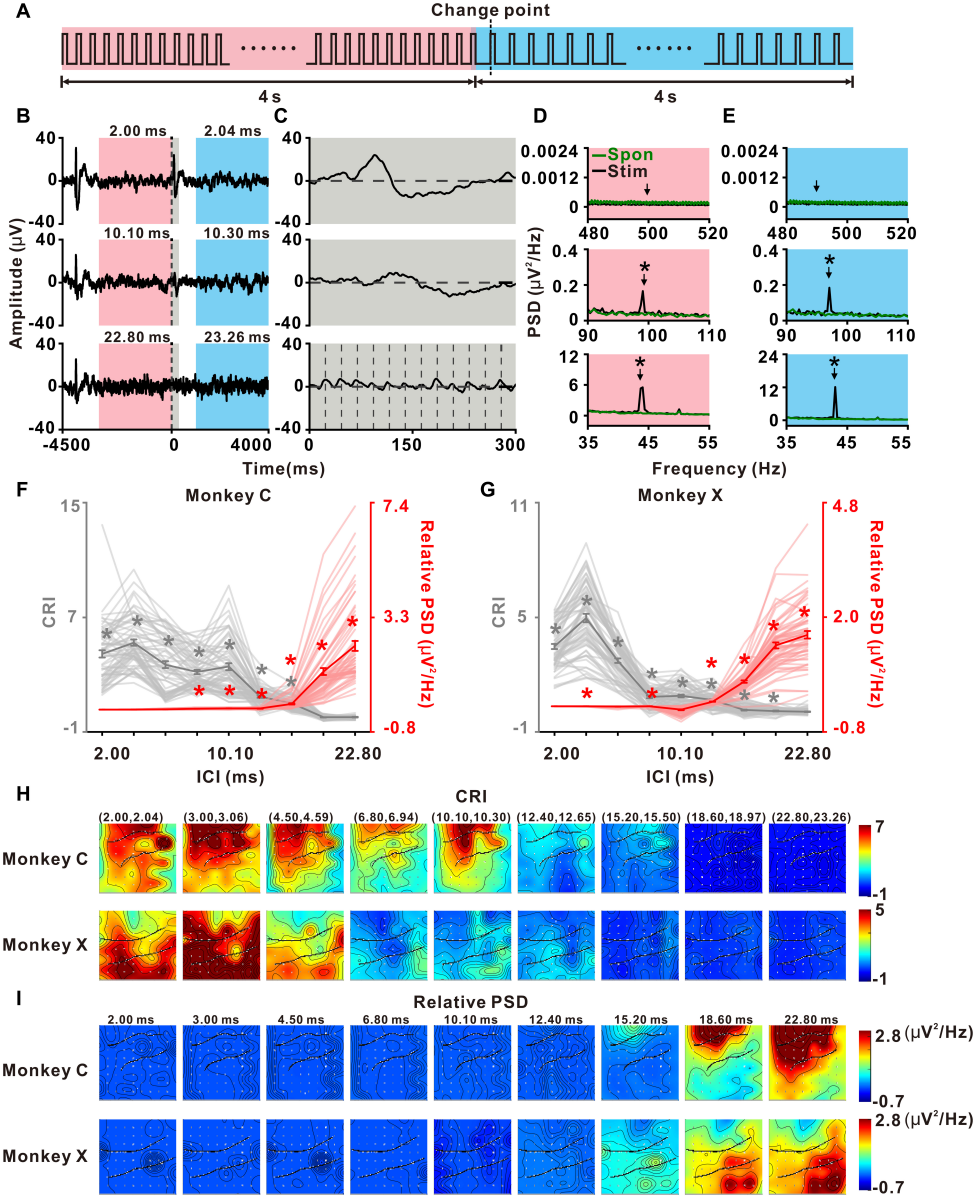
ECoG responses to transitional click train. (A) Structure of the transitional click train, composed of two 4-s regular click trains (i.e., click trains with constant ICIs). Each train starts with a specific ICI selected from 2, 10.1, and 22.8 ms (red), followed by another train whose ICI is increased to 1.02 times its initial value (blue). The dashed line indicates the transition point between the 2 trains. (B) Responses of a representative ECoG site to various transitional click trains with different representative ICI combinations. The zero point marks the transition, with the dashed line aligned accordingly. The red and blue backgrounds denote segments used for FFT analysis in (D) and (E). The gray background in (B) indicates the zoomed-in area shown in (C). (C) Zoomed-in view of the initial 300-ms post-transition from (B). Vertical dashed lines in the bottom plots indicate the onset of each individual click. (D and E) Black lines represent the spectrum in PSD obtained by FFT analysis of the segments indicated by red (D) and blue (E) backgrounds in (B). Green lines in each subplot represent spectra of the spontaneous period. Arrows indicate frequencies corresponding to the click rates within these segments. Asterisks indicate that the PSD at that frequency is significantly larger than its neighboring frequency (*P* < 0.001, one-tailed paired *t* test, FDR-corrected, 0.5-Hz bins) for the black line. No significant peak at the corresponding frequencies was detected for the spontaneous period in the green line in all subplots (*P* > 0.05, one-tailed paired *t* test, FDR-corrected, 0.5-Hz bins). (F and G) CRI (gray) and PSD [red, corresponding to the segment in red background in (B) and (D)] as a function of ICI for monkey C (F) and monkey X (G) across all tested ICI combinations. Individual light lines represent single recording sites, while the darker lines depict the average CRI values (gray) and PSD values (red). Error bars indicate SEM. ICIs with significant CRI or PSD across recording sites are marked with asterisks (*P < 0.05, rank-sum test). (H and I) Tonotopic distribution of CRI values (H) and relative PSD (I) across all tested ICI combinations (indicated at the top of each graph) for monkey C (top row of panels) and monkey X (bottom row of panels). In each graph, 2 lines denote the lateral sulcus (upper line) and the superior temporal sulcus (bottom line), and light gray dots mark the location of ECoG electrode sites.

To investigate neural signals related to pitch perception and distinguish them from signals tracking individual click, we conducted electrocorticography (ECoG) recordings in A1 of 2 rhesus monkeys (Fig. S1). We designed a transitional click-train paradigm by concatenating two 4-s regular click trains, each characterized by distinct ICIs (Fig. 1A). The first ICI (ICI_1_) were configured as 2, 10.1, and 22.8 ms (corresponding to 500, 99.01, and 43.86 Hz of repetition rate, respectively), while the second ICI (ICI_2_) was set at 1.02 times the value of ICI_1_ for each condition, resulting in 2% decrease in repetition rate (Fig. 1B). This transitional click train is referred to as Reg_ICI1-ICI2_. Figure 1B to E illustrates exemplar ICIs of 2.0 ms (500 Hz), 10.1 ms (99.01 Hz), and 22.8 ms (43.86 Hz), while the complete ICI set used for the tuning curves and tonotopic maps is shown in Fig. 1F to I.

The analysis of a representative ECoG channel reveals an onset response at the beginning of each click train, followed by neural adaptation, resulting in a reduced response to subsequent clicks (Fig. 1B). This adaptation is consistently observed across all tested conditions (Fig. 1B). Interestingly, at the transition point where a new click train with a different ICI is introduced, the previously adapted neural channel exhibited a distinct peak response, specifically for ICIs of 2 and 10.1 ms (corresponding to 500 and 99.01 Hz repetition rate, respectively) (Fig. 1C). Negligible change in response occurred when the ICI_1_ was 22.8 ms (43.86 Hz repetition rate). Further examination of the data reveals a pronounced change response within the initial 300 ms following the transition, as depicted in Fig. 1C. The change response is not merely a transient response due to the sudden change of the ICI, evidenced by the absence of a similar response to the insertion of a single different interval within a regular click train with constant ICI (top row of Fig. S2B), indicating that the change response we observed necessitates the temporal integration of multiple intervals (Fig. S2). Therefore, this change response likely signifies a shift in perception of pitch [34].

In addition to examining the change response, we also explored the neural response to individual clicks by analyzing synchronization patterns using fast Fourier transform (FFT) of signal segments. These segments are highlighted with a shadow background in Fig. 1B. They were analyzed before and after the transition in the transitional click train (black traces in Fig. 1D and E) alongside a spontaneous baseline immediately preceding stimulus onset (green traces in Fig. 1D and E). A detailed inspection shows that synchronization strength [measured with power spectral density (PSD) of corresponding repetition rate] increases as a function of the ICI length, as demonstrated by the progression from top to bottom in Fig. 1D and E. As expected, synchronization was not observed when the ICI was less than 3 ms (corresponding to repetition rates above 333.33 Hz; red traces in Fig. 1F and G), consistent with the known upper limit of phase locking in primate A1 [7]. Notably, we observed that in Reg_10.1-10.3_ condition, synchronization to individual clicks co-occurred with robust change responses, indicating that 2 distinct temporal scales—click-level synchronization and train-level synchronization—were simultaneously represented (Fig. 1C to E). We refer to this phenomenon as temporal integration during synchronization (TIDS), wherein neurons both phase-lock to rapid input events and integrate them into temporally structured percepts. This phenomenon suggests that the auditory cortex can integrate individual clicks while simultaneously synchronizing with them, thus representing 2 distinct temporal scales concurrently while ICI is in a proper range.

Next, we conducted a population analysis. To quantify the magnitude of the change response, we computed a change response index (CRI), defined as the normalized root mean square (RMS) amplitude of the change response (see Methods). Across both monkeys, CRI decreased monotonically with increasing ICI (gray color, Fig. 1F and G). Conversely, the synchronization strength (red color, Fig. 1F and G) shows clear increase with longer ICIs (*r* = 0.93, *P* < 0.001, Spearman’s rank correlation, Fig. 1F; *r* = 0.88, *P* < 0.01, Spearman’s rank correlation, Fig. 1G). These results were replicated when synchronization was quantified using the phase-locking factor (PLF; see Methods; Fig. S3A and B). In monkey C, ICIs ranging from 6.8 to 15.2 ms (repetition rates from 147.06 to 65.79 Hz) were associated with both significant change response and significant synchronization with individual clicks. In monkey X, this relationship extended to ICIs ranging from 12.4 to 18.6 ms (repetition rates from 80.65 to 53.76 Hz; Fig. 1F and G). This inverse relationship between CRI and synchronization strength as ICI increased was also evident in the tonotopic distribution of change responses and synchronization strength, where the areas responding shrank for CRI but expanded for synchronization strength as ICI increased (Fig. 1H and I and Fig. S3C for PLF). The opposite trends observed for the CRI and synchronization strength across ICIs highlight the distinct neuronal mechanisms underlying temporal integration and phase-locking. Robust temporal integration into click trains (as reflected in the change response) is favored at shorter ICIs, when the auditory system groups rapid clicks into a unified percept. In contrast, synchronization to individual clicks becomes dominant at longer ICIs, when the temporal spacing allows reliable phase-locking. These findings demonstrate that the auditory cortex dynamically shifts between integration and synchronization modes depending on the temporal characteristics of the stimulus, supporting efficient processing across multiple timescales.

Notably, we observed that TIDS, characterized by the simultaneous presence of click-level synchronization and robust train transition responses, emerge only for ICIs within an intermediate range (approximately 6 to 18 ms, corresponding to repetition rates of 166.67 to 55.56 Hz; Fig. 1F and G). At shorter ICIs (<3 ms, repetition rate > 333.33 Hz), the neural capacity for phase-locking to individual clicks is lost, and at longer ICIs (>20 ms, repetition rate < 50 Hz), temporal integration into a unified train object is reduced. Thus, TIDS is constrained to a “proper range” of ICIs in which both neural synchrony and integration are simultaneously supported by the underlying physiology.

**Fig. 2.**
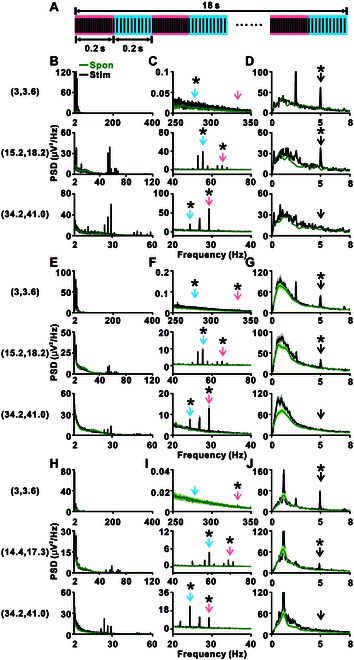
Synchronous tracking of individual clicks and click trains in ECoG signals. (A) An 18-s click train consisting of 2 short (0.2 s) alternating trains with different ICIs. The ICI combinations were chosen randomly from 3 groups: 3 to 3.6 ms, 15.2 to 18.2 ms, and 34.2 to 41 ms. Red backgrounds indicate trains with the shorter ICI, while blue backgrounds highlight those with the longer ICI. (B) Black lines represent the FFT spectra in PSD of an example recording site from monkey C for ICI combinations of 3 to 3.6 ms (top), 15.2 to 18.2 ms (middle), and 34.2 to 41 ms (bottom). Green lines in each subplot represent the spectra from the spontaneous baseline immediately preceding stimulus onset. (C and D) Zoomed-in views of (B) showing the synchronous response to individual clicks (C) and distinct click trains (D), respectively. (E) Aggregate FFT spectra in PSD from all recording sites in monkey C for the ICI combinations of 3 to 3.6 ms (top), 15.2 to 18.2 ms (middle), and 34.2 to 41 ms (bottom). Shaded areas indicate 2 SEM. (F and G) Detailed views of (E) illustrating responses to individual clicks (F) and click trains (G). (H to J) Arranged in the same format as (E) to (G) from monkey X for ICI combinations of 3 to 3.6 ms (top), 14.4 to 17.3 ms (middle), and 34.2 to 41 ms (bottom). Arrows mark frequencies corresponding to ICIs (C, F, and I) or trains (D, G, and J). Asterisks indicate that the PSD at that frequency is larger than its neighboring frequency (*P* < 0.001, one-tailed paired *t* test, FDR-corrected, 0.5-Hz bins) for the black line. No significant peak at the corresponding frequencies was detected for the spontaneous period in the green line in all subplots (*P* > 0.05, one-tailed paired *t* test, FDR-corrected, 0.5-Hz bins).

To directly assess the brain’s capacity to track temporal features at both fine and coarse scales, we designed a prolonged click-train stimulus (18 s) consisting of 2 short (0.2 s) alternating trains with distinct ICIs (Fig. 2A). To evoke a robust change response despite potential neural adaptation during sustained stimulation, we used a larger ICI ratio of 1.2 (rather than 1.02). We tested 3 pairs of ICIs in monkey C: (3, 3.6); (15.2, 18.2); (34.2, 41.0) ms. Figure 2B shows the FFT analysis of the response from one example channel during the presentation of the prolonged click train, with enlarged sections in Fig. 2C and D, corresponding to individual-click tracking and click-train tracking, respectively. Green traces denote spectra from the spontaneous baseline immediately preceding stimulus onset (Fig. 2C and D). Individual-click tracking depended on the ICI, with their corresponding frequencies indicated by arrows in Fig. 2C. No peak was detected for the ICI pair (3, 3.6) ms, but peaks were observed for ICI pairs (15.2, 18.2) ms and (34.2, 41.0) ms. As the click train switched every 200 ms, corresponding to 5 Hz, peaks were detected for ICI pairs (3, 3.6) ms and (15.2, 18.2) ms (Fig. 2D). Thus, for the ICI pair (15.2, 18.2) ms, the brain could track both individual clicks and click trains synchronously. Similar results were also found in the population data across all 64 recording channels (Fig. 2E to G). In monkey X, we also tested 3 ICI pairs: (3, 3.6) ms; (14.4, 17.3) ms; (34.2, 41.0) ms. For the ICI pair (14.4, 17.3) ms, the brain could track both individual clicks and click trains synchronously (Fig. 2H to J).

### Temporal structures across multiple scales with a click-train-based oddball

**Fig. 3.**
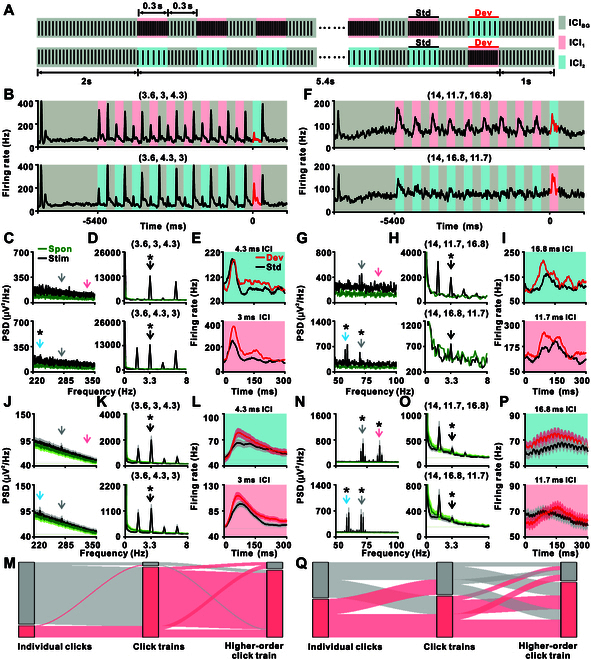
Neuronal responses in A1 to Click-Train-Based Oddball Paradigm. (A) Experimental setup of the Click-Train-Based Oddball Paradigm. The first sequence (top) features a background click train with a given ICI (ICI_BG_, gray background) for 2 s, followed by alternating 300-ms click trains with ICIs of ICI_1_ (red background) and ICI_BG_ for 5.4 s. Responses during this 5.4-s segment are analyzed for neuronal synchronization with clicks and alternating click trains. Each train with ICI_1_ appears 9 times before a new deviant train with ICI_2_ (blue background) is introduced, forming the sequence (ICI_BG_, ICI_1_, ICI_2_). The second set (bottom) reverses standard and deviant train roles while retaining the background train, creating the other matching oddball sequence (ICI_BG_, ICI_2_, ICI_1_). Red and black horizontal bars indicate standard and deviant periods for response comparison. (B to I) An example A1 neuron’s response to short ICIs (B to E) and long ICIs (F to I). (B) PSTHs of a representative neuron for sequences (3.6, 3, 4.3 ms; top) and (3.6, 4.3, 3 ms; bottom). Light gray, blue, and red backgrounds indicate click trains with ICIs of 3.6, 4.3, and 3 ms, respectively. (C and D) Black lines represent the spectra of the 5.4-s segment for individual click (C) and alternating click trains (D) in sequences (3.6, 4.3, 3 ms; top) and its counterpart (3.6, 3, 4.3 ms; bottom). Green lines in each subplot represent the spectra of the spontaneous period. Arrows highlight corresponding frequencies, and asterisks indicate that the PSD at that frequency is larger than its neighboring frequency (*P* < 0.001, one-tailed paired *t* test, FDR-corrected, 0.5-Hz bins) for the black line. No significant peak at the corresponding frequencies was detected for the spontaneous period in the green line in all subplots (*P* > 0.05, one-tailed paired *t* test, FDR-corrected, 0.5-Hz bins). (E) PSTHs for neuronal responses to click trains with ICIs of 4.3 ms (top) and 3 ms (bottom), comparing deviant (red) and the last standard (black) presentations. (F) PSTHs of the same neuron for sequences (14, 11.7, 16.8 ms; top) and (14, 16.8, 11.7 ms; bottom). Light gray, blue, and red backgrounds represent ICIs of 14, 16.8, and 11.7 ms, respectively. (G and H) Black lines represent the spectra for synchronization with individual clicks (G) and alternating click trains (H) in sequences (14, 11.7, 16.8 ms; top) and its counterpart (14, 16.8, 11.7 ms; bottom). Green lines in each subplot represent the spectra of the spontaneous period. Arrows mark corresponding frequencies, and asterisks indicate that the PSD at the frequency is larger than its neighboring frequency (*P* < 0.001, one-tailed paired *t* test, FDR-corrected, 0.5-Hz bins) for the black line. No significant peak at the corresponding frequencies was detected for the spontaneous period in the green line in all subplots (*P* > 0.05, one-tailed paired *t* test, FDR-corrected, 0.5-Hz bins). (I) PSTHs for neuronal responses to click trains with ICIs of 11.7 ms (top) and 16.8 ms (bottom), distinguishing between deviant (red) and the last standard (black) presentations. (J to Q) Population neuronal responses in A1 (*n* = 127) for short ICIs (J to M) and long ICIs (N to Q). For short ICIs, (J) to (L) are arranged in the same format as (C) to (E). Shaded area covers 2 SEM, with the line illustrating the average of all neurons. (M) Sankey diagram depicting the flow of significant proportions from first level (individual clicks) to third level (higher-order click train) in sequences (3.6, 4.3, 3) ms and its counterpart. For simplicity, only the background ICI_BG_ is considered for the first level, and both blocks are grouped for the first and second levels. Significant proportions are marked in red (*P* < 0.05, one-tailed paired *t* test), while nonsignificant proportions are in gray. For the third level, red indicates a proportion with a positive CSI, with other proportions shown in gray. For long ICIs, (N) to (P) are arranged in the same format as (G) to (I), and (Q) is arranged as (M), presenting the results for sequences (14, 11.7, 16.8) ms and its counterpart.

After our initial investigation of tracking multiple temporal scales using ECoG signals, we turned our attention to the neuronal substrate and so we conducted single-unit recording in A1. To advance our understanding of auditory temporal processing across various scales, we introduced a novel click-train-based oddball paradigm (Fig. 3A). This paradigm involved 2 sets of continuous click trains.

In the first set (the top row of Fig. 3A), a background click train with a specific ICI (ICI_BG_, indicated by gray background) played continuously for 2 s. This was followed by alternating click trains with ICIs of ICI_1_ (indicated by red background) and ICI_BG_ every 300 ms for the next 5.4 s. Each train was presented 9 times before introducing a new train with ICI_2_ (indicated by blue background) as a deviant train. The set was labeled as (ICI_BG_, ICI_1_, ICI_2_). In the second set (the bottom row of Fig. 3A), we reversed the roles of the standard and deviant trains while maintaining the background train, creating the extended train (ICI_BG_, ICI_2_, ICI_1_). This experimental setup enabled us to assess the ability of the brain to track individual clicks (first level), click trains (second level), and higher-order click train (third level) across 3 different temporal scales. We applied this click-train-based oddball paradigm using 2 types of ICIs—short (3.6 ms as ICI_BG_) and long (14 ms as ICI_BG_)—to test the paradigm’s effectiveness under different temporal conditions. Monitoring individual clicks and click trains was facilitated through FFT analysis of the 5.4-s alternating click trains within the block. The mismatch signal was derived by comparing the deviant train (indicated by the red horizontal bar in Fig. 3A) in one set to the same train presented as the standard (indicated by the black horizontal bar in Fig. 3A) in the alternate set.

Figure 3B to I illustrates the responses of a representative A1 neuron to this click-train-based oddball paradigm. For shorter ICI set (3.6, 3, 4.3) ms and their counterpart (3.6, 4.3, 3) ms, the peri-stimulus time histogram (PSTH) of the neuronal response displayed a peak at the beginning of the extended click train, followed by a return to baseline. The response then oscillated during the alternating presentations of the background train and the standard train, with a robust response observed for the deviant train (Fig. 3B, red trace). An in-depth analysis revealed a lack of significant synchronization with individual clicks (indicated by arrows in the Fig. 3C), but a peak around 3.3 Hz suggested synchronization with distinct click trains (*P* < 0.001 for both blocks, one-tailed paired *t* test, FDR-corrected; Fig. 3D). Further, the response was significantly stronger when the deviant train was presented as compared to when it served as the standard stimulus (*P* < 0.05 for both conditions, *t* test; Fig. 3E).

For longer ICIs (14, 11.7, 16.8) ms and their counterpart (14, 16.8, 11.7) ms, the PSTH of the same example neuron showed oscillatory responses during the alternating presentations of the background train and the standard train, followed by a robust response to the deviant train (Fig. 3F, red trace). Detailed analysis revealed that for the ICIs of 14, 11.7, and 16.8 ms, there was no significant synchronization to individual clicks at 71.4 and 85.5 Hz (*P* > 0.05, one-tailed paired *t* test, FDR-corrected; top panel in Fig. 3G). However, significant synchronization was observed for ICIs of 14, 16.8, and 11.7 ms (*P* < 0.05, one-tailed paired *t* test, FDR-corrected; bottom panel in Fig. 3G). Moreover, a significant peak around 3.3 Hz was observed under some conditions (*P* < 0.001, one-tailed paired *t* test, FDR-corrected; top panel in Fig. 3H), indicating synchronization with the train object as a whole. Notably, the response was significantly stronger when the train was presented as the deviant stimulus as compared to when it was presented as the standard stimulus (*P* < 0.05 for both conditions, *t* test; Fig. 3I). During the spontaneous period, no peaks were detected either near ~3.3 Hz or at the individual-click rates (green traces in Fig. 3C and D and F and G; *P* > 0.05 for all conditions, one-tailed paired *t* test, FDR-corrected).

In the neuronal population analysis for short ICIs, no significant synchronization with individual clicks was detected either for the (3.6, 3, 4.3) condition or for the (3.6, 4.3, 3) condition (*P* > 0.05 for all conditions, one-tailed paired *t* test, FDR-corrected; Fig. 3J). However, synchronization with distinct click trains was consistently robust and highly significant (*P* < 0.001, one-tailed paired *t* test, FDR-corrected; Fig. 3K), and the mismatch signal was also significant for both types of trains (*P* < 0.001, paired *t* test; Fig. 3L). More specifically, out of 127 neurons analyzed, 15 exhibited synchronizations with individual clicks, 119 demonstrated significant synchronization with distinct click trains, and 116 of these neurons showed mismatch signals (Fig. 3M). For longer ICIs, we observed significant synchronization with both individual clicks (*P* < 0.001, one-tailed paired *t* test, FDR-corrected; Fig. 3N) and distinct click trains (*P* < 0.001, one-tailed paired *t* test, FDR-corrected; Fig. 3O) for both ICI sets. On the other hand, we observed significant differences between the deviant and standard trains for the train with 16.8-ms ICI (*P* < 0.001, one-tailed paired *t* test, top panel in Fig. 3P), but no significant difference for the train with 11.7-ms ICI (*P* > 0.05, one-tailed paired *t* test; bottom panel in Fig. 3P). Out of the 127 neurons analyzed for longer ICIs, 66 demonstrated synchronization with individual clicks, 52 showed significant synchronization with distinct click trains, and 95 exhibited mismatch signals (Fig. 3Q). These findings emphasize the remarkable ability of neuronal populations to effectively track multiple temporal scales.

### Exploring multiple temporal scales at the neuronal level in MGB

Having established that neurons in A1 can track multiple temporal scales very efficiently, we investigated whether or not the MGB neurons show similar characteristics to test if this ability was an emergent property of A1 or inherited from the thalamus. Hence, we conducted single-unit recordings in the MGB using an identical experimental protocol as detailed above for A1 (Fig. 3A).

**Fig. 4.**
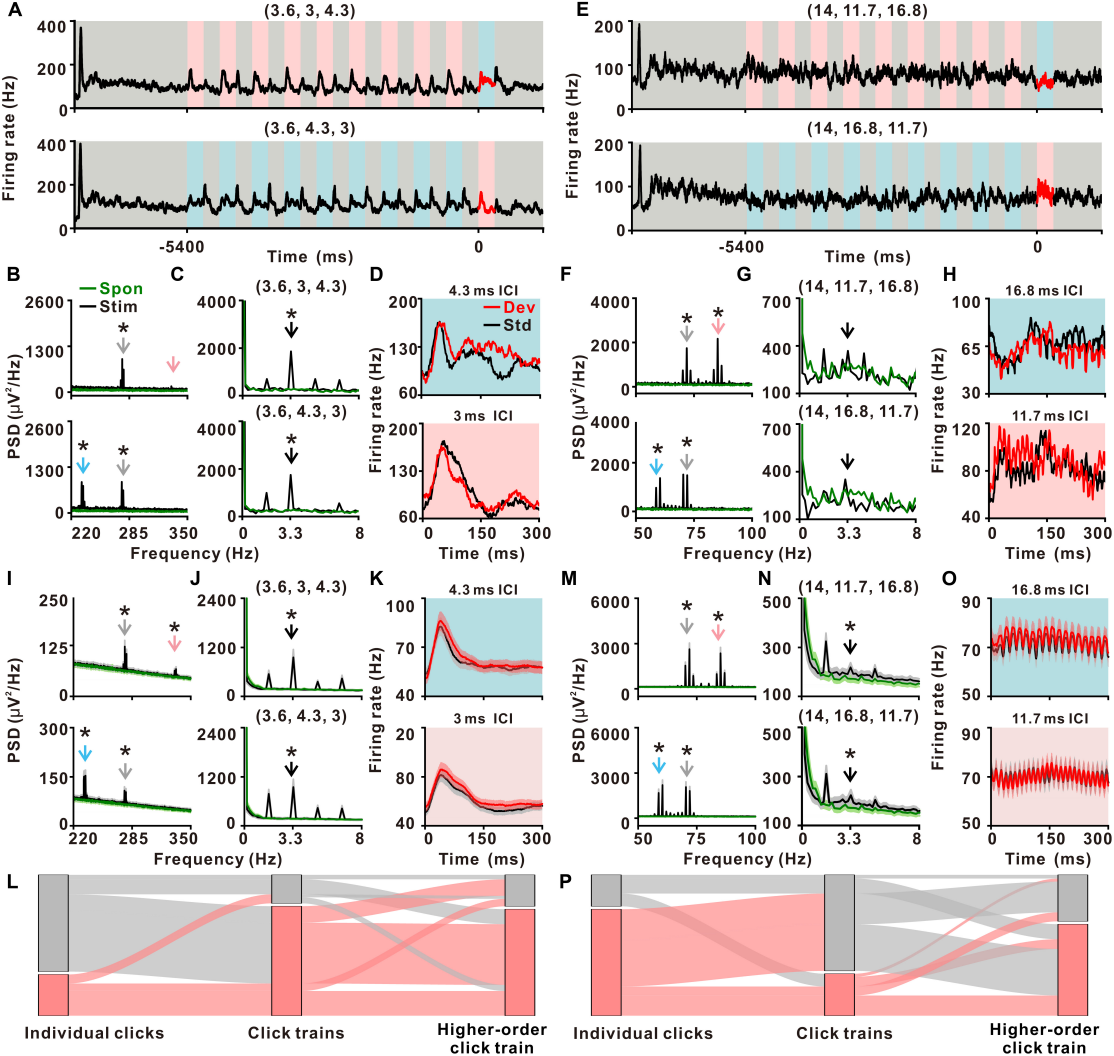
Neuronal responses in MGB to the Click-Train-Based Oddball Paradigm. (A to H) An example MGB neuron’s responses to short ICIs (A to D) and long ICIs (E to H), same as Fig. 3B to I. (I to P) Population neuronal responses in MGB (*n* = 184) for short ICIs (I to L) and long ICIs (M to P), same as Fig. 3J to Q.

For short ICIs (3.6, 3, 4.3) ms and their counterpart (3.6, 4.3, 3) ms, the PSTH of neuronal responses in one example MGB neuron showed a peak at the start of the extended train, followed by a return to baseline (Fig. 4A). The responses then oscillated in synchrony with the alternating presentation of the background train and standard train (Fig. 4A). Moreover, we observed significant synchronization with both individual clicks (*P* < 0.001 at 277.8 Hz for both blocks, one-tailed paired *t* test, FDR-corrected; Fig. 4B) and distinct click trains at around 3.3 Hz (*P* < 0.001 for both blocks, one-tailed paired *t* test, FDR-corrected; Fig. 4C). For the 4.3-ms train, responses to deviant trains were significantly greater than to standard trains (*P* < 0.05, one-tailed paired *t* test, top panel in Fig. 4D), whereas no significant difference was observed for the 3-ms train (*P* = 0.61, one-tailed paired *t* test; bottom panel in Fig. 4D). For longer ICIs (14, 11.7, 16.8) ms and their counterpart (14, 16.8, 11.7) ms, there was significant synchronization with individual clicks (*P* < 0.001 for both blocks, one-tailed paired *t* test, FDR-corrected; Fig. 4F), but not for the distinct click trains represented at around 3.3 Hz (*P* > 0.05 for both blocks, one-tailed paired *t* test, FDR-corrected; Fig. 4G). Similarly, we did not detect significant difference in responses to deviant and standard trains for both train types (*P* > 0.05 for both blocks, one-tailed paired *t* test; Fig. 4H). During the spontaneous period, no peaks were detected either near ~3.3 Hz or at the individual-click rates (green traces in Fig. 4B and C and F and G; *P* > 0.05 for all conditions, one-tailed paired *t* test, FDR-corrected).

The population-level analysis revealed that MGB neurons significantly synchronized with both individual clicks (*P* < 0.001 for both blocks, one-tailed paired *t* test, FDR-corrected; Fig. 4I) and distinct click trains (*P* < 0.001 for both blocks, one-tailed paired *t* test, FDR-corrected; Fig. 4J) for short ICI conditions. Responses to deviant and standard trains look similar, but the deviant response was significantly stronger than the standard one (*P* < 0.05 for both types of trains, one-tailed paired *t* test; Fig. 4K). Out of 184 neurons analyzed, 55 exhibited synchronizations with individual clicks, 146 showed significant synchronization with distinct click trains, and 142 of these neurons demonstrated significant mismatch signals for short ICIs (Fig. 4L). This response pattern was consistent for longer ICIs as well (Fig. 4M to P).

### Comparing hierarchical temporal processing between A1 and MGB

**Fig. 5.**
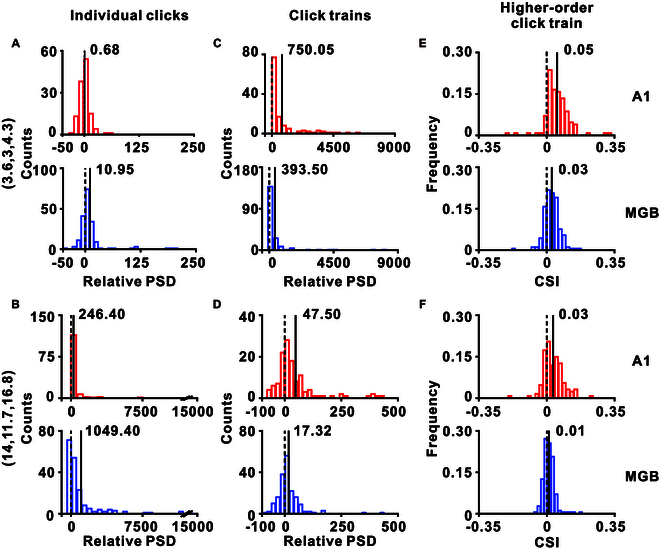
Comparative analysis between A1 and MGB responses across temporal scales. (A and B) Distribution of relative PSD at corresponding frequencies for the first level (individual clicks) in responses to click-train-based oddball stimuli with short (A) and long (B) ICIs. The A1 responses (red) and MGB responses (blue) are compared. (C and D) Distribution of relative PSD at corresponding frequencies for the second level (click trains) in responses to click-train-based oddball stimuli with short (C) and long (D) ICIs, again comparing A1 (red) and MGB (blue). (E and F) Distribution of the CSI value for the third level (higher-order click train) in responses to click-train-based oddball stimuli with short (E) and long (F) ICIs for A1 (red) and MGB (blue). In each histogram, one vertical dashed line is located at *X* = 0; the vertical solid line, accompanied by a floating number, indicates the mean value.

Next, we precisely evaluated these responses in MGB and A1 and this direct comparison revealed notable differences. While synchronization with individual clicks was higher in the MGB than in the A1 for both short and long ICIs (*P* < 0.05 for both conditions, *t* test; Fig. 5A and B), synchronization with distinct click trains was more pronounced in the A1 for both ICIs (*P* < 0.05 for both conditions, *t* test; Fig. 5C and D). These above results were replicated when synchronization was quantified using PLF (Fig. S4). Regarding the mismatch signal, the CSI was greater in the A1 than in the MGB for both short and long ICIs (*P* < 0.05 for both conditions, *t* test; Fig. 5E and F). These findings suggest that while both the A1 and MGB neurons are capable of synchronizing with multiple temporal scales, they prioritize different aspects of temporal information processing. Thus, A1 neurons seem more specialized for tracking the click trains (second level) or the higher-order click train (third level) in temporal patterns, whereas MGB neurons exhibit a stronger synchronization with individual clicks (first level).

### Tracking multiple temporal scales in humans

**Fig. 6.**
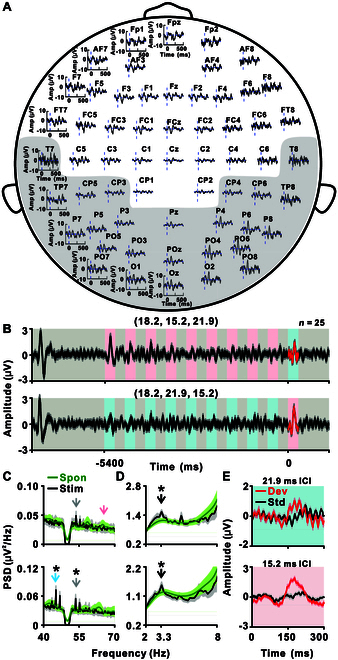
Electrophysiological responses to the click-train-based oddball paradigm in human. (A) Onset responses of all EEG channels to stimulus sequences (18.2, 15.2, 21.9 ms) from a subject. Each subplot corresponds to one channel at its respective scalp location, with identical axis settings. The vertical dashed line marks the onset of the train stimuli. The channels in the light gray background were selected for further analysis. (B) Mean waveform of EEG signal across all the selected channels for sequences (18.2, 15.2, 21.9 ms; top) and (18.2, 21.9, 15.2 ms; bottom). Light gray, blue, and red backgrounds represent ICIs of 18.2, 21.9, and 15.2 ms, respectively. Shaded gray area covers 2 SEM (*n* = 25). (C and D) Black lines represent the spectra of the 5.4-s segment for synchronization with individual click (C) and click trains (D) in sequences (18.2, 15.2, 21.9 ms; top) and its counterpart (bottom). Shaded area covers 2 SEM (*n* = 25). Green lines in each subplot represent the spectra of the spontaneous period. Arrows mark the corresponding frequencies, and asterisks indicate that the PSD at that frequency is larger than its neighboring frequency (*P* < 0.001, one-tailed paired *t* test, FDR-corrected, 0.5-Hz bins) for the black line. No significant peak at the corresponding frequencies was detected for the spontaneous period in the green line in all subplots (*P* > 0.05, one-tailed paired *t* test, FDR-corrected, 0.5-Hz bins). (E) Average EEG response to click trains with ICIs of 21.9 (top) and 15.2 ms (bottom), comparing deviant (red) and the last standard (black) presentations. Shaded gray area covers 2 SEM (*n* = 25).

Considering the fundamental role of temporal integration in the brain [35,36], its relevance to many psychiatric diseases [26,29–32], and the clinical relevance of oddball paradigms [37–39], the click-train-based oddball paradigm serves as a promising tool for diagnosis. To explore the potential for clinical application of this paradigm, we conducted 64-channel EEG recordings in 25 subjects using a paradigm structurally identical to those used in the monkey experiments and illustrated in Fig. 3A, but with species-specific parameterization. The human sequence employed (18.2, 15.2, 21.9 ms) and its counterpart, whereas the monkey sequence used (14, 11, 16.8 ms) and its counterpart. A representative example shows a robust auditory onset response at the beginning of the extended click train in the temporal, parietal, and occipital lobes (Fig. 6A). By focusing on 25 channels within these regions (covered by light gray background in Fig. 6A), we captured detailed responses to the click-train-based oddball paradigm. The results revealed distinct oscillatory patterns and robust responses to deviant stimuli (Fig. 6B). Critically, human EEG data revealed population-level neural synchronization with both individual clicks and click trains, mirroring the synchronization and temporal integration observed in macaque ECoG and single-unit recordings. At the population level, we observed significant synchronization with both individual clicks [ICIs (18.2, 15.2, 21.9) ms: *P* < 0.001 and *P* > 0.05 at 55.0 and 65.8 Hz, respectively; *P* < 0.001 for ICIs (18.2, 21.9, 15.2) ms, one-tailed paired *t* test, FDR-corrected; Fig. 6C] and click trains (*P* < 0.001, one-tailed paired *t* test, FDR-corrected; Fig. 6D). During the spontaneous period, no peaks were detected either near ~3.3 Hz or at the individual-click rates (green traces in Fig. 6C and D; *P* > 0.05 for all conditions, one-tailed paired *t* test, FDR-corrected). To further validate the reliability of these synchronization effects in human EEG data, we analyzed trial-to-trial phase consistency using the PLF. As shown in Fig. S5, strong PLF peaks were observed at both the single-click (40 to 80 Hz) and click-train (~3.3 Hz) frequencies, confirming consistent phase-locking across trials and complementing the FFT/PSD analyses. Additionally, responses to deviant stimuli were consistently more robust than those to standard stimuli across both trains (*P* < 0.05 for both click trains, paired *t* test; Fig. 6E), resembling the deviance detection observed in primate cortex (Fig. 3). Figure S6 presents topographical distributions of deviant and standard responses across all EEG channels. These findings highlight tracking of temporal structures across multiple scales with nonlinguistic structured stimuli and the potential of the click-train-based oddball paradigm for use in clinical settings to aid diagnosis.

## Discussion

The current study demonstrates that the A1 neurons possess the ability to track temporal structures at multiple scales. Here, we have designed a novel train object-based oddball paradigm that allows the investigation of auditory responses at multiple time scales. Using ECoG in monkeys and EEG in human subjects, we observed distinct neural responses to click-train sequence transitions (Fig. 1), highlighting the capacity of the brain to simultaneously represent various temporal scales (Fig. 1C to E). Further, an experiment with prolonged click trains demonstrated the brain’s ability to concurrently track individual click and the overall structure of click trains (Fig. 2). By using the click-train-based oddball paradigm, we noted significantly stronger responses in A1 to deviant trains compared to standard trains, showcasing an enhanced capacity for recognizing temporal patterns (Fig. 3). This ability was also evident in the auditory thalamus (Fig. 4), but the MGB neurons exhibited a preference for tracking individual click, while cortical A1 neurons preferred the entire click trains (Fig. 5). In human subjects, EEG recordings confirmed synchronization with both individual clicks and click trains (Fig. 6C and D), and more pronounced responses to deviant trains (Fig. 6E), suggesting the paradigm’s potential for clinical applications. Collectively, our findings demonstrate that the auditory brain has an extraordinary ability to discern and process intricate auditory temporal patterns, offering significant insights for both basic neuroscience and potential clinical applications.

### Multiple temporal scale tracking from mesoscopic to macroscopic levels

The exploration of multiple temporal scales in auditory processing has traditionally focused on macroscopic levels, employing methods like EEG and functional magnetic resonance imaging (fMRI) [2,4,40–42]. Although EEG and fMRI have substantially advanced our understanding of the capacity of the brain in temporal processing, these studies generally focus on the response of larger brain areas or networks, leaving the role of individual neurons or small neuronal populations less explored. On the other hand, traditional studies often utilized language-based stimuli to unravel the auditory system’s temporal dynamics [2,4,42], leveraging the intricacies of spoken language. Our study introduces a new design, the click-train-based oddball paradigm, offering a much-refined tool for auditory temporal processing research. By extending the use of click trains—traditionally employed in auditory research [6,7,10,43–45]—to encompass multiple temporal scales, we investigate temporal processing reminiscent of linguistic structures, but without the complexities of language. This novel approach enabled us to examine temporal scales from mesoscopic to macroscopic levels, including both animal and human studies.

Our ECoG recordings in rhesus monkeys have unveiled a sophisticated capacity for temporal processing in A1, as evidenced by the synchronization with individual click and the change response in train transitions (Figs. 1C to E and 2). Crucially, the change response, underpinned by temporal integration (Fig. S2), indicates that trains with varying ICIs are perceived as distinct auditory objects, a notion further corroborated by the mismatch signals in the click-train-based oddball paradigm (Fig. 3A). Previously, it has been argued that click trains with an ICI under approximately 33 ms often exhibit pitch-like qualities [10], suggesting that our transitional click-train paradigm introduces a switch in perception while maintaining neuronal adaptation through continuous click-train presentation (Fig. 1). Here, the change response may represent a shift in pitch perception and the synchronization with the click trains in prolonged click trains potentially signals alterations in pitch perception (Fig. 2). Interestingly, while temporal integration is critical in auditory processing, only recent research has identified signals related to the conceptual grouping in spoken language [2], such as combining syllables into phrases and phrases into sentences. Here, the change response provides a novel and convenient measure for investigating temporal integration, applicable in both animal and human studies without the necessity of language comprehension. Furthermore, the synchronization with click trains (Fig. 2) and mismatch signal (Fig. 6) suggests an inherent ability of the auditory brain to organically group clicks into coherent auditory objects during continuous stimulation.

Our single-unit recordings fill the gap between mesoscopic and macroscopic levels, offering direct observations of how individual neurons and neuronal assemblies in areas like A1 and the MGB respond to multiple temporal scales (Figs. 3 and 4). For example, while EEG and fMRI have suggested a hierarchical organization in temporal capacity in spoken language [4,46–48], our single-unit data offers a neuronal correlate for this hierarchy, showing how different auditory regions contribute uniquely to processing of various temporal scales (Fig. 5).

### Mechanism underlying tracking of multiple scales of temporal structures

Our study employed the click-train-based oddball paradigm, presenting continuous clicks, yet it encapsulates 3 scales of temporal information (Fig. 3A): the individual clicks (first level), the click trains (second level), and higher-order click train (third level). We compared responses between A1 and MGB at the neuronal level concerning these 3 temporal scales.

At the first level (individual clicks), with an ICI as low as around 3 ms, A1 showed poor synchronization, as indicated by the low peak in its corresponding FFT analysis (Fig. 3C and J). However, under the same stimulus conditions, neurons in the MGB demonstrated stronger synchronization (Fig. 4B and I). This difference regarding the first level persisted even with longer ICIs (around 15 ms), where both A1 (Fig. 3N) and MGB (Fig. 4M) exhibited significant synchronization with individual click, but the synchronization was more pronounced in the MGB (Fig. 5A and B). These findings align with previous research [49,50], suggesting that MGB neurons synchronize with individual clicks at higher repetition rates compared to A1 neurons. This difference implies a shift in coding strategy from temporal to rate coding in auditory processing of the first level [50,51].

When considering the second level (click train), both A1 and MGB demonstrated significantly stronger synchronization with shorter ICIs compared to longer ones (Fig. 5C and D). This pattern suggests that click trains with short ICIs are more effectively perceived as unified auditory objects, resulting in more pronounced change responses, as shown in Fig. 1. Regular click trains, particularly those with shorter ICIs, are often perceived as having pitch-like qualities [11,52,53]. Traditional theories, also supported by computational models, have attributed this perception of pitch to processing at the basilar membrane [53]. Given the MGB’s high capacity for stimulus synchronization [49,50], one might expect it to show stronger synchronization if pitch perception were solely reliant on the basilar membrane. However, in contrast to first-level processing, the A1 exhibited significantly more pronounced synchronization with click trains than the MGB, suggesting a superior capacity for temporal integration in the A1. This observation leads to an alternative hypothesis regarding the origin of pitch perception in click trains. Namely, it may be rooted in temporal integration processes within the A1. This hypothesis warrants further investigation, particularly through studies using prolonged alternating click-train paradigms. Moreover, the differences between the A1 and MGB in terms of TIDS are also noteworthy. The neuronal responses in A1 to prolonged click trains with continuous transitions not only involved synchronization with individual clicks but also displayed robust overall change responses to successive transitions of distinct click trains (Fig. 3K and O). This pattern indicates the presence of TIDS at the neuronal level within the A1. In contrast, while MGB neurons synchronized with individual clicks, their overall change responses to transitions of click trains was markedly weaker (Fig. 4J and N). These findings highlight an advanced capacity of A1 neurons in temporal integration and its essential role in processing and interpreting complex auditory temporal structures. Our findings indicate that TIDS is selectively present only within a “proper range” of ICIs (approximately 6 to 18 ms), where both phase-locking and temporal integration mechanisms are active. This “proper range” reflects the biophysical limits of auditory cortical neurons: At ICIs shorter than ~3 ms, phase-locking is lost, while at longer ICIs (>20 ms), temporal integration into a cohesive auditory object diminishes. Importantly, the third and longest timescale of our paradigm (higher-order click train) elicits a different type of neural response: a robust mismatch or change signal, rather than ongoing synchronization. This dissociation demonstrates that distinct neural mechanisms support auditory processing at each temporal level.

Finally, the third-level mismatch signal in our click-train-based oddball paradigm presents a compelling aspect, traditionally examined at the neuronal level through SSA [15–17,23,24,54–56], which has been extensively studied across various parts of the auditory pathway, including the A1 [57–65], MGB [24,56,63,66–70], inferior colliculus (IC) [18,71–77], and cochlear nucleus [73]. Typically, these studies have utilized pure tones to generate oddball stimuli, with previous data suggesting that SSA is mediated by adaptation in narrowly tuned inputs, a concept supported by computational modeling [78] and the discovery of synaptic SSA [55]. However, more recent studies have proposed that SSA is predominantly a predictive process [22,79–82]. This theory finds support in our findings from the click-train-based oddball paradigm. We observed minimal SSA in the MGB (Fig. 4K and O), while the SSA in A1 was pronounced, accompanied by a substantial change response for deviant transitions (Fig. 3L and P). Notably, our paradigm is characterized by the continuous presentation of identical clicks, challenging the notion that the selective feedforward adaptation model can solely account for the observed SSA. This observation leads us to consider that SSA in our paradigm may arise more from a predictive process than solely through adaptation mechanisms, as suggested in other studies [16–25]. One limitation of our oddball paradigm is that the deviant train was always presented after a fixed number of standard trains within each block. This deterministic structure may have allowed participants (or underlying neural circuits) to anticipate the deviant’s occurrence, potentially influencing response magnitude or timing. While this approach facilitated statistical power and cross-condition comparisons, future studies should consider introducing temporal variability in the presentation of the deviant to better control for predictive effects and more closely align with classical oddball paradigms.

### Potential clinical implications of the click-train-based oddball paradigm

The click-train-based oddball paradigm offers a novel framework for probing temporal integration and its neural correlates [83]. Because temporal integration deficits have been documented in several psychiatric and neurological conditions [26,29–31], this approach could provide a mechanistically grounded extension of existing clinical paradigms. Importantly, our present findings are limited to healthy populations and experimental models; thus, any translational application will require further validation.

In particular, the click-train-based oddball paradigm provides a novel approach to study the MMN signal. MMN is already a valuable tool in clinical applications, used for assessing auditory processing disorders [37], cognitive impairment [37], and various conditions such as schizophrenia [38,39] and Alzheimer’s disease [37]. It is also employed in pediatric research to detect language and learning difficulties in children [84] and investigate the effects of drugs [85]. The MMN signal obtained through this click-train-based oddball paradigm (Fig. 6E) could potentially become a valuable biomarker for broader clinical diagnostics, aiding in the assessment and diagnosis of these conditions. Nevertheless, while these findings are promising, it is important to emphasize that our human EEG data reflect population-level activity rather than single-neuron precision. Consequently, further research is required to directly link these population-level EEG markers to specific underlying neural mechanisms. The translational potential of this approach will depend on future studies employing source-localized EEG, intracranial recordings, or cross-species comparisons to clarify the relationship between scalp signals and neuronal dynamics.

In summary, our investigation significantly deepens the understanding of auditory temporal processing by offering a detailed examination at the neuronal level. This approach not only complements but also enriches the insights gained from EEG and fMRI studies. By extending the scope from human to animal models, from linguistic to nonlinguistic sounds, and from macroscopic to microscopic perspectives, our study has uncovered some foundational mechanisms underlying the ability of the auditory system to code complex temporal patterns, thereby broadening our comprehension of auditory temporal processing.

### Technical limitations and cross-species considerations

Our conclusions should be interpreted with due consideration for several limitations, particularly those inherent to nonhuman primate and human studies. While the inclusion of 4 rhesus macaques constitutes an ethically sound and technically robust cohort within the domain of nonhuman primate electrophysiology, it remains modest when compared to rodent studies, which routinely employ substantially larger sample sizes. Although we maximized statistical power through within-subject designs and high neuronal yields, future investigations involving larger primate cohorts—or meta-analytic approaches—will be important for a more comprehensive assessment of inter-individual variability.

A further limitation concerns the spatial and mechanistic resolution of our recordings. The present ECoG and neuronal data lack laminar specificity and do not provide causal insights into underlying circuit mechanisms. Indeed, the synaptic pathways supporting the proposed “temporal integration during synchronization” remain to be elucidated. Rodent models will be essential for experimentally testing the mechanistic predictions generated by our primate data, as they allow for larger cohorts, cell type-specific tools, and more invasive techniques such as optogenetics and high-density laminar probes. For example, they may help determine whether corticofugal feedback enhances temporal object representations in the MGB or the IC.

Addressing these limitations through cross-species, multi-level approaches will not only enhance the generalizability of our findings but also clarify which elements of hierarchical temporal processing are specific to primates and which reflect conserved mammalian principles. Nevertheless, despite these constraints, we believe that the present study offers a meaningful and timely contribution to the field and provides a solid foundation for future work.

## Methods

### Subjects and surgical procedures

Our research involved 4 male rhesus monkeys, identified as M, C, X, and Z, aged 7 to 8 years and weighing between 5.5 and 8 kg. We performed ECoG recordings with monkeys X and C, and extracellular recordings with monkeys M and Z. The experimental protocols were rigorously in line with the guidelines set by the State Council of the People’s Republic of China (GB 14925-2010) and received approval from the Bioethics Committee of Zhejiang University (approval no. ZJU20210078), ensuring that the animals received comprehensive care and ethical treatment throughout the study duration. A dedicated team conducted daily health and well-being checks on the monkeys, enhancing their living conditions with a variety of toys and preferred food items in their 0.74-m^3^ home cages to promote exploratory behavior [86].

For implantation of the headpost and ECoG array, we adopted sterile surgical techniques. Premedication was administered using ketamine (50 mg/kg) and medetomidine (0.03 mg/kg). The monkeys were then intubated and connected to an artificial respirator (A.D.S.1000, Engler Engineering Corp., FL) to maintain their body temperature at a stable 37 °C, using an electric heating mat. Vital signs such as oxygen saturation, heart rate, and end-tidal CO₂ were continuously monitored (Surgi Vet, Smiths Medical PM Inc., London, UK) to precisely adjust anesthesia levels as needed.

The surgical procedure entailed securing the skull in a stereotactic frame (Narishige, Tokyo, Japan). Following lidocaine administration, skin and muscle tissue at the target site were meticulously excised. A titanium headpost (Gray Matter Research, MT) was then anchored to the skull using dental resin and bone screws. Craniotomy and durotomy were performed under the guidance of a microscope (Ophthalmo-Stativ S22, Carl Zeiss Inc., Oberkochen, Germany), equipped with a complementary metal-oxide semiconductor (CMOS) color camera (TS-CA-130MIII, MeCan Imaging Inc., Saitama, Japan). After positioning the ECoG array, the surgical site was sealed with the dura mater, bone flap, and skin, and the exposed area of the skull was covered with dental resin. To promote recovery and minimize infection risk, postoperative care included administering ketoprofen for 3 days and antibiotics for a week.

### Sound stimulation

Experiments were conducted in a soundproof environment. Acoustic stimuli were digitally synthesized using a computer-controlled Auditory Workstation [RZ6, Tucker-Davis Technologies (TDT)] at a 100-kHz sampling rate, and played back through a high-fidelity speaker (LS50, KEF, UK), which was placed on the right side, contralateral to the recording side. Sound pressure levels were meticulously calibrated using a ^1^/_4_-inch condenser microphone (Brüel & Kjær 4954, Nærum, Denmark) and a PHOTON/RT analyzer (Brüel & Kjær, Nærum, Denmark) to ensure precision in stimulus delivery.

To evaluate neuronal responses, we focused on 2 primary metrics: the frequency response area (FRA) and the characteristic frequency (CF). A series of tones ranging from 0.2 to 16.1 kHz, in 26 logarithmic steps, with 100-ms duration and a 5-ms rise–fall time, were presented. Tone intensity varied from 0- to 70-dB sound pressure level (SPL) in 10-dB increments. Each combination of frequency and intensity was presented 5 times, separated by 500-ms inter-stimulus intervals.

Our study utilized click trains comprising monophasic electrical pulses of condensation polarity, each with a duration of 0.2 ms (200 μs). Each click-train-based stimulus was presented at an average intensity of 60-dB SPL throughout the stimulus duration, calibrated at the center of the head. We used 3 complementary paradigms to probe temporal processing at different levels, as detailed below.

1. Transitional Click Train Paradigm: To investigate neural adaptation and change detection at temporal boundaries, we developed a transitional click-train paradigm, Reg_ICI1-ICI2_. This paradigm consisted of two 4-s regular click trains with different ICIs; the first ICI (ICI_1_) ranged from 2 ms to 22.8 ms, while the second (ICI_2_) was set at 1.02 times ICI_1_ (Fig. 1). Each condition was presented 40 times for recording.

To determine if the change response in the transitional click train was due to temporal integration or a mere reaction to abrupt ICI changes, we inserted 1, 2, 4, and 8 intervals with inserted ICI (ICI_inserted_) at the 4-s mark of an 8-s click train with a constant ICI (ICI_constant_); ICI_inserted_ was 1.02 times the ICI_constant_. The corresponding transitional click train served as the control in this analysis (Fig. S2). Each condition was presented 40 times for recording.

2. Prolonged Alternating Click Train Paradigm: To test simultaneous neural tracking of both fine (individual-click level) and coarse (click-train level) temporal structure, we designed an extended 18-s click train (Fig. 2A). This included 2 alternating short trains, each 0.2 s long, with 2 different ICIs. The longer interval was set at 1.2 times the length of the shorter one. This paradigm was used to reveal the brain’s ability to synchronously track events at both the individual-click and click-train timescales. The Prolonged Alternating Click Train was presented 40 times for each recording.

3. Click-Train-Based Oddball Paradigm: To examine synchronous processing across 3 distinct temporal scales (individual clicks, click trains, and higher-order click train), we introduced a novel click-train-based oddball paradigm (Fig. 3A). This involved continuous click trains in 2 configurations: an initial 2-s consistent background click train (ICI_BG_, gray background in Fig. 3A), followed by alternating click trains marked by ICI_1_ (red background in Fig. 3A) and ICI_BG_ every 300 ms. This sequence was repeated 9 times (for 5.4 s) followed by a deviant train with ICI_2_ (blue background in Fig. 3A). In the counterpart setup, we reversed the roles of the standard and deviant trains while maintaining the background train constant. Each of the 2 configurations was presented randomly, with 40 repetitions for both monkey experiments and human experiments.

A summary of the stimulus designs is provided in Table 1.

**Table 1.** Experimental parameters of click-train paradigms

Paradigm	ICIs	Stimulus duration	Inter-trial interval	Trial number	Related figure	Total time
Transitional Click Train (monkey, ECoG)	ICI_1_: [2, 3, 4.5, 6.8, 10.1, 12.4, 15.2, 18.6, 22.8] ms ICI_2_: 1.02 * ICI_1_	8 s	5 s	40	Fig. 1	78 min
Prolonged Alternating Click Train (monkey, ECoG)	ICI_1_: [3,14.4 (monkey X)/15.2 (monkey C), 18.6, 22.8, 34.2] ms ICI_2_: 1.2 * ICI_1_	18 s	6 s	40	Fig. 2	80 min
Click-Train-Based Oddball (monkey, SU)	ICI_BG_, ICI_1_, ICI_2_: ICIs (3.6, 3, 4.3) ms ICIs (14, 11.7, 16.8) ms	8.4 s	1.6 s	40	Figs. 3–5	26 min 40 s
Click-Train-Based Oddball (human, EEG)	ICI_BG_, ICI_1_, ICI_2_: ICIs (18.2, 21.9, 15.2) ms	8.4 s	1.6 s	40	Fig. 6	13 min 20 s

### ECoG recording

We implanted a 64-channel electrode array (E64-2500-50-200, NeuroNexus Technologies Inc., Michigan, USA) subdurally over the primary auditory cortex (A1) of 2 rhesus macaques, as depicted in Fig. 1A. These electrodes, with a diameter of 200 μm, had an inter-electrode distance of 2.5 mm and featured gold surfaces, on a 2 × 2 cm polyimide substrate. The electrode impedances, assessed at 1 kHz, ranged from 20 to 50 kilohms. For accurate placement, preoperative MRI scans (MAGNETOM 7T, Siemens Healthcare, Erlangen, Germany) were used to identify target locations and determine the appropriate sizes for craniotomies. A gold reference electrode was strategically positioned near the ECoG array within the subdural space, aimed toward the dura. Lead wires from both the ECoG array and the reference electrode were connected to micro-connectors (ZIF-Clip 64, TDT, Alachua, FL), which were then encased within a titanium chamber firmly attached to the skull with resin. ECoG signals were amplified and digitized at 1.22 kHz using a differential amplifier (PZ5, TDT), and band-pass filtered between 0.5 and 600 Hz (second-order biquad filter, TDT). The processed signals were then stored on hard drives for subsequent detailed analysis.

Recorded ECoG signals segmented from 2 s before stimulus onset to 2 s after stimulus offset. To mitigate interference from nonphysiological sources, independent component analysis (ICA) was employed using the FieldTrip toolbox [87].

### Extracellular recording

In each session, we utilized multi-site linear electrodes (S-probe, Plexon, USA): 16 contacts for the A1 and 32 for the MGB. These electrodes were inserted into the brain based on MRI structural guidance through 26-gauge transdural guide tubes (Fig. S7) and advanced using a remote-controlled microdrive (FHC, ME). Neural activity was recorded at a sampling rate of 12.2 kHz and amplified 20,000-fold using a differential amplifier (RZ5, TDT). Neuronal spike trains were extracted from raw waveforms and sorted offline using Kilosort, which applies a digital Butterworth high-pass filter with a cutoff frequency of 150 Hz. Manual curation was subsequently performed in Phy [88,89].

In total, we recorded 127 neurons in the A1 and 184 neurons in the MGB, using the various click-train sequences explained above. For our recordings within A1, we partitioned the area based on its tonotopic organization as determined by CFs. To achieve this, we recorded the responses of 50 additional neurons to pure tones.

### Human EEG experiment

We recruited 25 volunteers (mean age 25.2 ± 3.1 years; 14 females, 11 males), all with self-reported normal hearing. The study was meticulously conducted in compliance with ethical standards, receiving approval from the Ethics Committee of the Women’s Hospital School of Medicine, Zhejiang University (IRB-20230131-R). Each participant provided informed written consent before participating in the study. During the experimental sessions, participants were comfortably positioned with head support in an acoustically isolated room. Click sounds, each with a duration of 0.2 ms, were synthesized using MATLAB R2021a (The MathWorks Inc., Natick, MA, USA) on a computer equipped with a high-fidelity sound card (Creative, AE-7). These clicks were produced with a sampling rate of 384 kHz and 32-bit precision, then broadcasted through a stereo speaker system (Golden Field M23), ensuring the precise presentation of the click-train-based oddball paradigm to participants under rigorously controlled conditions.

Continuous EEG data were recorded using a 64-channel NeuSenW system (Neuracle, China) at a sampling rate of 1 kHz. The reference electrode was positioned at the center of the head near Cz, and the ground electrode was placed on the forehead. The raw EEG data were band-pass filtered between 2 and 400 Hz using a digital Butterworth filter implemented in the FieldTrip toolbox. Additionally, a 50-Hz notch filter was applied to eliminate any potential electric interference. Furthermore, ICA was applied to remove potential eye artifacts.

### Data analysis and statistics

PSTHs for discrete spike data were computed using a 1.5-ms bin width and a 1-ms step. These PSTHs were treated equivalently to continuous data (i.e., ECoG and EEG) in the following analyses.

To assess the synchronized tracking of individual clicks (first level) and click trains (second level), we applied FFT analysis to signal segments. Single-sided PSD was computed using FFT with a rectangular window, such that the squared magnitude of the Fourier coefficients (normalized by sampling rate and signal length) provided an estimate of power distribution across positive frequencies:

$$PSD\left(f\right)=\frac{2{\left|A\left(f\right)\right|}^{2}}{N\times f_{s}}$$ (1)

Here, *A*(*f*) denotes single-sided amplitude at frequency *f*. *N* is the number of samples, and *fs* is the sample rate. This measure was used to assess whether neural responses exhibited distinct peaks at specific frequency components.

To detect spectral peaks, we utilized one-tailed paired *t* test to determine if responses in a frequency bin (0.5 Hz width) were significantly stronger than the adjacent lower frequency bin (0.5 Hz width). FDR correction using the Benjamini–Hochberg method was applied for multiple comparisons across frequency bins. Peak PSD was quantified as the difference between a given frequency bin and its neighboring bin. The relative PSD at a specific frequency was calculated as the difference of the peak PSD between the stimulus period and the spontaneous period (Figs. 1F, G, and I and 5A to D). The spontaneous period was defined as a 2-s window preceding stimulus onset for ECoG recordings in the Transitional Click Train Paradigm and a 1.8-s window in the Prolonged Alternating Click Train Paradigm, a 1-s window for single-unit recordings, and a 0.5-s window for EEG recordings.

While PSD analysis provided a robust means of identifying spectral power at stimulus-related frequencies, it primarily reflects amplitude changes and does not directly capture the degree of phase consistency across trials. To complement the PSD results and to more directly quantify temporal precision and reliability of neural synchronization, we therefore examined phase-locking using a wavelet-based approach. Phase-locking across trials was quantified using a complex Morlet wavelet framework, following Tallon-Baudry et al. [90]. For each center frequency *f*_0_, a complex Morlet wavelet was constructed with a spectral bandwidth determined by the ratio *f*_0_/*σ_f_* = 7. The temporal standard deviation *σ_t_* = 1/(2*πσ_f_*), and the wavelet was truncated at ±3*σ_t_* to ensure energy concentration. Single-trial signals were convolved with the wavelet, yielding complex-valued time series of the same length as the original data. Each time series was normalized to unit vectors to isolate phase information. The PLF at each time point was computed as the modulus of the across-trial average of these unit vectors:

$$PLF\left(t,\;f\right)=\left|\frac{1}{N}\sum_{n=1}^{N}e^{i\phi_{n}\left(t,f\right)}\right|$$ (2)

where *N* is the number of trials and $\phi_{n}\left(t,f\right)$ is the instantaneous phase of trial *n* at time *t* and frequency *f*. Specifically, $\phi_{n}\left(t,f\right)$ was computed using continuous ECoG/EEG data (Figs. S3 and S5) or PSTHs of discrete spike data (Fig. S4). Statistical significance of phase concentration was evaluated using Rayleigh’s test [91]. For each time point and frequency, the test statistic was computed as

$$R=\left|\sum_{n=1}^{N}e^{{\mathrm{i\phi}}_{n}\left(t,f\right)}\right|$$ (3)

$$Z=\frac{R^{2}}{N}$$ (4)

The corresponding *P* value was approximated by

$$p\approx e^{-Z}\left(1+\frac{2Z-Z^{2}}{N}\right),$$ (5)

which provides a large-sample correction for finite *N*. For each frequency and channel, a mean PLF value (Figs. S3 and S4) was derived by averaging across time points with significant phase-locking (*P* < 0.05) [91].

PLF was applied in both the Transitional Click Train Paradigm (Fig. S3) and the Click-Train-Based Oddball Paradigm (Figs. S4 and S5), using neural data obtained from ECoG, EEG, and single-unit recordings. Frequency ranges were matched to the stimulation paradigm: 40 to 80 Hz with 0.1-Hz resolution for individual clicks (Fig. S5A), and 2 to 5 Hz with 0.01-Hz resolution for click trains (Fig. S5B).

In the transitional click-train paradigm, we quantified the CRI to characterize the response magnitude as follows:

$$\mathrm{CRI}=\frac{\mathrm{RM}S_{\text{Change}}-\mathrm{RM}S_{\text{Control}}}{\mathrm{RM}S_{\text{Change}}+\mathrm{RM}S_{\text{Control}}}$$ (6)

Here, RMS_Change_ refers to the RMS calculated from 0 to 300 ms relative to the transition point of the train stimulation (i.e., the occurrence of the first interval for the second train), and RMS_Control_ represents the RMS within the previous 300-ms window.

For the third level, we used the common SSA index (CSI) to quantify the degree of SSA in the oddball paradigm [18,57,63,92,93]. CSI was calculated according to the following formula:

$$\mathrm{CSI}=\frac{d_{1}+d_{2}-s_{1}-s_{2}}{d_{1}+d_{2}+s_{1}+s_{2}}$$ (7)

Here, *s_i_* and *d_i_* (*i* = 1, 2) indicate standard and deviant responses. *s_1_* and *d_1_* refer to the response to ICI_1_ in the condition of (ICI_BG_, ICI_1_, ICI_2_) and (ICI_BG_, ICI_2_, ICI_1_), respectively; *s_2_* and *d_2_* refer to the response to ICI_2_ in the condition of (ICI_BG_, ICI_2_, ICI_1_) and (ICI_BG_, ICI_1_, ICI_2_). The response measurement used in this calculation was the average firing rate in the 0- to 300-ms window relative to the onset of either the deviant train or the last standard train.

The significance of relative PSD and CRI among 64 channels in ECoG recordings was calculated using the Wilcoxon rank-sum test (Fig. 1F and G). For the significance tests for the target frequency peaks in the response spectrum, we conducted paired one-sided *t* test with FDR correction. Additionally, we used the 2-tailed independent *t* test to compare the A1 and MGB (Fig. 5).

## Data Availability

The data are available at the following link: https://doi.org/10.57760/sciencedb.30601.

## References

[1] Gao R, van den Brink RL, Pfeffer T, Voytek B. Neuronal timescales are functionally dynamic and shaped by cortical microarchitecture. eLife. 2020;9: Article 61277. 10.7554/eLife.61277 PMC7755395 33226336

[2] Ding N, Melloni L, Zhang H, Tian X, Poeppel D. Cortical tracking of hierarchical linguistic structures in connected speech. Nat Neurosci. 2016;19(1):158–164. 26642090 10.1038/nn.4186 PMC4809195

[3] Chait M, Greenberg S, Arai T, Simon JZ, Poeppel D. Multi-time resolution analysis of speech: Evidence from psychophysics. Front Neurosci. 2015;9:214. 26136650 10.3389/fnins.2015.00214 PMC4468943

[4] Lerner Y, Honey CJ, Silbert LJ, Hasson U. Topographic mapping of a hierarchy of temporal receptive windows using a narrated story. J Neurosci. 2011;31(8):2906–2915. 21414912 10.1523/JNEUROSCI.3684-10.2011 PMC3089381

[5] Caucheteux C, Gramfort A, King JR. Evidence of a predictive coding hierarchy in the human brain listening to speech. Nat Hum Behav. 2023;7:430–441. 36864133 10.1038/s41562-022-01516-2 PMC10038805

[6] Lu T, Liang L, Wang X. Temporal and rate representations of time-varying signals in the auditory cortex of awake primates. Nat Neurosci. 2001;4(11):1131–1138. 11593234 10.1038/nn737

[7] Steinschneider M, Reser DH, Fishman YI, Schroeder CE, Arezzo JC. Click train encoding in primary auditory cortex of the awake monkey: Evidence for two mechanisms subserving pitch perception. J Acoust Soc Am. 1998;104(5):2935–2955. 9821339 10.1121/1.423877

[8] Oshurkova E, Scheich H, Brosch M. Click train encoding in primary and non-primary auditory cortex of anesthetized macaque monkeys. Neuroscience. 2008;153(4):1289–1299. 18423884 10.1016/j.neuroscience.2008.03.030

[9] Bendor D, Wang X. Differential neural coding of acoustic flutter within primate auditory cortex. Nat Neurosci. 2007;10(6):763–771. 17468752 10.1038/nn1888

[10] Krumbholz K, Patterson RD, Pressnitzer D. The lower limit of pitch as determined by rate discrimination. J Acoust Soc Am. 2000;108:1170–1180. 11008818 10.1121/1.1287843

[11] Yost WA, Mapes-Riordan D, Shofner W, Dye R, Sheft S. Pitch strength of regular-interval click trains with different length “runs” of regular intervals. J Acoust Soc Am. 2005;117(5):3054–3068. 15957774 10.1121/1.1863712 PMC2709838

[12] Fitzgerald K, Todd J. Making sense of mismatch negativity. Front Psych. 2020;11:468. 10.3389/fpsyt.2020.00468 PMC7300203 32595529

[13] Garrido MI, Kilner JM, Stephan KE, Friston KJ. The mismatch negativity: A review of underlying mechanisms. Clin Neurophysiol. 2009;120(3):453–463. 19181570 10.1016/j.clinph.2008.11.029 PMC2671031

[14] Naatanen R, Paavilainen P, Rinne T, Alho K. The mismatch negativity (MMN) in basic research of central auditory processing: A review. Clin Neurophysiol. 2007;118(12):2544–2590. 17931964 10.1016/j.clinph.2007.04.026

[15] Khouri L, Nelken I. Detecting the unexpected. Curr Opin Neurobiol. 2015;35:142–147. 26318534 10.1016/j.conb.2015.08.003

[16] Malmierca MS, Auksztulewicz R. Stimulus-specific adaptation, MMN and predictive coding. Hear Res. 2021;399: Article 108076. 10.1016/j.heares.2020.108076 32933789

[17] Song P, Zhai Y, Yu X. Stimulus-specific adaptation (SSA) in the auditory system: Functional relevance and underlying mechanisms. Neurosci Biobehav Rev. 2023;149: Article 105190. 37085022 10.1016/j.neubiorev.2023.105190

[18] Malmierca MS, Cristaudo S, Pérez-González D, Covey E. Stimulus-specific adaptation in the inferior colliculus of the anesthetized rat. J Neurosci. 2009;29(17):5483–5493. 19403816 10.1523/JNEUROSCI.4153-08.2009 PMC2715893

[19] Carbajal GV, Casado-Román L, Malmierca MS. Two prediction error systems in the nonlemniscal inferior colliculus: “Spectral” and “nonspectral”. J Neurosci. 2024;44(23): Article e1420232024. 38627089 10.1523/JNEUROSCI.1420-23.2024 PMC11154860

[20] Casado-Román L, Carbajal GV, González DP, Malmierca MS. Prediction error signaling explains neuronal mismatch responses in the medial prefrontal cortex. PLOS Biol. 2020;18(12): Article e3001019. 33347436 10.1371/journal.pbio.3001019 PMC7785337

[21] Pérez-González D, Lao-Rodríguez AB, Aedo-Sánchez C, Malmierca MS. Acetylcholine modulates the precision of prediction error in the auditory cortex. eLife. 2024;12: Article RP91475. 38241174 10.7554/eLife.91475 PMC10942646

[22] Gong Y, Song P, du X, Zhai Y, Xu H, Ye H, Bao X, Huang Q, Tu Z, Chen P, et al. Neural correlates of novelty detection in the primary auditory cortex of behaving monkeys. Cell Rep. 2024;43(3): Article 113864. 38421870 10.1016/j.celrep.2024.113864

[23] Zhai YY, Sun ZH, Gong YM, Tang Y, Yu X. Integrative stimulus-specific adaptation of the natural sounds in the auditory cortex of the awake rat. Brain Struct Funct. 2019;224(5):1753–1766. 31004193 10.1007/s00429-019-01880-2

[24] Rui YY, He J, Zhai YY, Sun ZH, Yu X. Frequency-dependent stimulus-specific adaptation and regularity sensitivity in the rat auditory thalamus. Neuroscience. 2018;392:13–24. 30248436 10.1016/j.neuroscience.2018.09.015

[25] Xu X-X, Zhai Y-Y, Kou X-K, Yu XJN. Adaptation facilitates spatial discrimination for deviant locations in the thalamic reticular nucleus of the rat. Neuroscience. 2017;365:1–11. 28942322 10.1016/j.neuroscience.2017.09.022

[26] Su L, Wyble B, Zhou LQ, Wang K, Wang YN, Cheung EFC, Bowman H, Chan RCK. Temporal perception deficits in schizophrenia: Integration is the problem, not deployment of attentions. Sci Rep. 2015;5:9745. 25940093 10.1038/srep09745 PMC4419531

[27] Todd J, Michie PT, Schall U, Karayanidis F, Yabe H, Näätänen R. Deviant matters: Duration, frequency, and intensity deviants reveal different patterns of mismatch negativity reduction in early and late schizophrenia. Biol Psychiatry. 2008;63(1):58–64. 17585889 10.1016/j.biopsych.2007.02.016

[28] Näätänen R, Kähkönen S. Central auditory dysfunction in schizophrenia as revealed by the mismatch negativity (MMN) and its magnetic equivalent MMNm: A review. Int J Neuropsychopharmacol. 2009;12(1):125–135. 18771603 10.1017/S1461145708009322

[29] Stevenson RA, Siemann JK, Schneider BC, Eberly HE, Woynaroski TG, Camarata SM, Wallace MT. Multisensory temporal integration in autism spectrum disorders. J Neurosci. 2014;34(3):691–697. 24431427 10.1523/JNEUROSCI.3615-13.2014 PMC3891950

[30] Nakano T, Ota H, Kato N, Kitazawa S. Deficit in visual temporal integration in autism spectrum disorders. Proc Biol Sci. 2010;277(1684):1027–1030. 19955150 10.1098/rspb.2009.1713 PMC2842756

[31] Panagiotidi M, Overton PG, Stafford T. Multisensory integration and ADHD-like traits: Evidence for an abnormal temporal integration window in ADHD. Acta Psychol. 2017;181:10–17. 10.1016/j.actpsy.2017.10.001 29024843

[32] Tokushige SI, Terao Y, Matsuda S, Furubayashi T, Sasaki T, Inomata-Terada S, Yugeta A, Hamada M, Tsuji S, Ugawa Y. Does the clock tick slower or faster in Parkinson’s disease?—Insights gained from the synchronized tapping task. Front Psychol. 2018;9:1178. 30050482 10.3389/fpsyg.2018.01178 PMC6051396

[33] Xu H, Huang Q, Song P, Chen Y, Li Q, Zhai Y, du X, Ye H, Bao X, Mehmood I, et al. EEG neural indicator of temporal integration in the human auditory brain with clinical implications. Commun Biol. 2025;8(1):1109. 40715543 10.1038/s42003-025-08540-8 PMC12297471

[34] Song P, Xu H, Ye H, du X, Zhai Y, Bao X, Huang Q, Mehmood I, Tanigawa H, Niu W, et al. Temporal merging into pitch with click train in the macaque auditory cortex. Natl Sci Rev. 2025;12(6): Article nwaf026. 40475067 10.1093/nsr/nwaf026 PMC12139000

[35] Mauk MD, Buonomano DV. The neural basis of temporal processing. Annu Rev Neurosci. 2004;27:307–340. 15217335 10.1146/annurev.neuro.27.070203.144247

[36] D’Argembeau A, Jeunehomme O, Majerus S, Bastin C, Salmon E. The neural basis of temporal order processing in past and future thought. J Cogn Neurosci. 2015;27(1):185–197. 24960045 10.1162/jocn_a_00680

[37] Iyer PM, Mohr K, Broderick M, Gavin B, Burke T, Bede P, Pinto-Grau M, Pender NP, McLaughlin R, Vajda A, et al. Mismatch negativity as an indicator of cognitive sub-domain dysfunction in amyotrophic lateral sclerosis. Front Neurol. 2017;8:395. 28861032 10.3389/fneur.2017.00395 PMC5559463

[38] Avissar M, Xie S, Vail B, Lopez-Calderon J, Wang Y, Javitt DC. Meta-analysis of mismatch negativity to simple versus complex deviants in schizophrenia. Schizophr Res. 2018;191:25–34. 28709770 10.1016/j.schres.2017.07.009 PMC5745291

[39] Umbricht D, Krljes S. Mismatch negativity in schizophrenia: A meta-analysis. Schizophr Res. 2005;76:1–23. 15927795 10.1016/j.schres.2004.12.002

[40] Chaudhuri R, Knoblauch K, Gariel MA, Kennedy H, Wang XJ. A large-scale circuit mechanism for hierarchical dynamical processing in the primate cortex. Neuron. 2015;88(2):419–431. 26439530 10.1016/j.neuron.2015.09.008 PMC4630024

[41] Chen J, Hasson U, Honey CJ. Processing timescales as an organizing principle for primate cortex. Neuron. 2015;88(2):244–246. 26494274 10.1016/j.neuron.2015.10.010

[42] Luo H, Poeppel D. Cortical oscillations in auditory perception and speech: Evidence for two temporal windows in human auditory cortex. Front Psychol. 2012;3:170. 22666214 10.3389/fpsyg.2012.00170 PMC3364513

[43] Brugge JF, Nourski KV, Oya H, Reale RA, Kawasaki H, Steinschneider M, Howard MA III. Coding of repetitive transients by auditory cortex on Heschl’s gyrus. J Neurophysiol. 2009;102(4):2358–2374. 19675285 10.1152/jn.91346.2008 PMC2775384

[44] Nourski KV, Brugge JF. Representation of temporal sound features in the human auditory cortex. Rev Neurosci. 2011;22(2):187–203. 21476940 10.1515/RNS.2011.016

[45] Nourski KV, Brugge JF, Reale RA, Kovach CK, Oya H, Kawasaki H, Jenison RL, Howard MA III. Coding of repetitive transients by auditory cortex on posterolateral superior temporal gyrus in humans: An intracranial electrophysiology study. J Neurophysiol. 2013;109(5):1283–1295. 23236002 10.1152/jn.00718.2012 PMC3602837

[46] Norman-Haignere SV, Long LK, Devinsky O, Doyle W, Irobunda I, Merricks EM, Feldstein NA, McKhann GM, Schevon CA, Flinker A, et al. Multiscale temporal integration organizes hierarchical computation in human auditory cortex. Nat Hum Behav. 2022;6(3):455–469. 35145280 10.1038/s41562-021-01261-y PMC8957490

[47] Stigliani A, Weiner KS, Grill-Spector K. Temporal processing capacity in high-level visual cortex is domain specific. J Neurosci. 2015;35(36):12412–12424. 26354910 10.1523/JNEUROSCI.4822-14.2015 PMC4563034

[48] Farbood MM, Heeger DJ, Marcus G, Hasson U, Lerner Y. The neural processing of hierarchical structure in music and speech at different timescales. Front Neurosci. 2015;9:157. 26029037 10.3389/fnins.2015.00157 PMC4429236

[49] Wang X, Lu T, Bendor D, Bartlett E. Neural coding of temporal information in auditory thalamus and cortex. Neuroscience. 2008;157(2):484–494. 19143093 10.1016/j.neuroscience.2008.07.050

[50] Bartlett EL, Wang X. Neural representations of temporally modulated signals in the auditory thalamus of awake primates. J Neurophysiol. 2007;97(2):1005–1017. 17050830 10.1152/jn.00593.2006

[51] Gao X, Wehr M. A coding transformation for temporally structured sounds within auditory cortical neurons. Neuron. 2015;86(1):292–303. 25819614 10.1016/j.neuron.2015.03.004 PMC4393373

[52] Gutschalk A, Patterson RD, Scherg M, Uppenkamp S, Rupp A. Temporal dynamics of pitch in human auditory cortex. NeuroImage. 2004;22(2):755–766. 15193604 10.1016/j.neuroimage.2004.01.025

[53] Balaguer-Ballester E, Denham SL, Meddis R. A cascade autocorrelation model of pitch perception. J Acoust Soc Am. 2008;124(4):2186–2195. 19062858 10.1121/1.2967829

[54] Gong Y, Zhai Y, du X, Song P, Xu H, Zhang Q, Yu X. Cross-modal interaction and integration through stimulus-specific adaptation in the thalamic reticular nucleus of rats. Neurosci Bull. 2022;38(7):785–795. 35212974 10.1007/s12264-022-00827-8 PMC9276886

[55] Zhai YY, Auksztulewicz R, Song PR, Sun ZH, Gong YM, du XY, He J, Yu X. Synaptic adaptation contributes to stimulus-specific adaptation in the thalamic reticular nucleus. Neurosci Bull. 2020;36(12):1538–1541. 32557078 10.1007/s12264-020-00536-0 PMC7719132

[56] Yu XJ, Xu XX, He S, He J. Change detection by thalamic reticular neurons. Nat Neurosci. 2009;12(9):1165–1170. 19684591 10.1038/nn.2373

[57] Ulanovsky N, Las L, Nelken I. Processing of low-probability sounds by cortical neurons. Nat Neurosci. 2003;6:391–398. 12652303 10.1038/nn1032

[58] Szymanski FD, Garcia-Lazaro JA, Schnupp JWH. Current source density profiles of stimulus-specific adaptation in rat auditory cortex. J Neurophysiol. 2009;102(3):1483–1490. 19571199 10.1152/jn.00240.2009

[59] Von Der Behrens W, Bäuerle P, Kössl M, Gaese BH. Correlating stimulus-specific adaptation of cortical neurons and local field potentials in the awake rat. J Neurosci. 2009;29(44):13837–13849. 19889995 10.1523/JNEUROSCI.3475-09.2009 PMC6666711

[60] Farley BJ, Quirk MC, Doherty JJ, Christian EP. Stimulus-specific adaptation in auditory cortex is an NMDA-independent process distinct from the sensory novelty encoded by the mismatch negativity. J Neurosci. 2010;30(49):16475–16484. 21147987 10.1523/JNEUROSCI.2793-10.2010 PMC6634869

[61] Taaseh N, Yaron A, Nelken I. Stimulus-specific adaptation and deviance detection in the rat auditory cortex. PLOS ONE. 2011;6(8): Article e23369. 21853120 10.1371/journal.pone.0023369 PMC3154435

[62] Nieto-Diego J, Malmierca MS. Topographic distribution of stimulus-specific adaptation across auditory cortical fields in the anesthetized rat. PLOS Biol. 2016;14(3): Article e1002397. 26950883 10.1371/journal.pbio.1002397 PMC4780834

[63] Antunes FM, Nelken I, Covey E, Malmierca MS. Stimulus-specific adaptation in the auditory thalamus of the anesthetized rat. PLOS ONE. 2010;5: Article e14071. 21124913 10.1371/journal.pone.0014071 PMC2988819

[64] Fishman YI, Steinschneider M. Searching for the mismatch negativity in primary auditory cortex of the awake monkey: Deviance detection or stimulus specific adaptation? J Neurosci. 2012;32(45):15747–15758. 23136414 10.1523/JNEUROSCI.2835-12.2012 PMC3641775

[65] Wang F, Liu J, Zhang J. Early postnatal noise exposure degrades the stimulus-specific adaptation of neurons in the rat auditory cortex in adulthood. Neuroscience. 2019;404:1–13. 30742959 10.1016/j.neuroscience.2019.01.064

[66] Anderson LA, Christianson GB, Linden JF. Stimulus-specific adaptation occurs in the auditory thalamus. J Neurosci. 2009;29(22):7359–7363. 19494157 10.1523/JNEUROSCI.0793-09.2009 PMC6666468

[67] Bäuerle P, von der Behrens W, Kössl M, Gaese BH. Stimulus-specific adaptation in the gerbil primary auditory thalamus is the result of a fast frequency-specific habituation and is regulated by the corticofugal system. J Neurosci. 2011;31(26):9708–9722. 21715636 10.1523/JNEUROSCI.5814-10.2011 PMC6623171

[68] Antunes FM, Malmierca MS. An overview of stimulus-specific adaptation in the auditory thalamus. Brain Topogr. 2014;27(4):480–499. 24343247 10.1007/s10548-013-0342-6

[69] Duque D, Malmierca MS, Caspary DM. Modulation of stimulus-specific adaptation by GABAA receptor activation or blockade in the medial geniculate body of the anaesthetized rat. J Physiol. 2014;592(4):729–743. 24099802 10.1113/jphysiol.2013.261941 PMC3934711

[70] Richardson BD, Hancock KE, Caspary DM. Stimulus-specific adaptation in auditory thalamus of young and aged awake rats. J Neurophysiol. 2013;110(8):1892–1902. 23904489 10.1152/jn.00403.2013 PMC3798939

[71] Zhao L, Liu Y, Shen L, Feng L, Hong B. Stimulus-specific adaptation and its dynamics in the inferior colliculus of rat. Neuroscience. 2011;181:163–174. 21284952 10.1016/j.neuroscience.2011.01.060

[72] Ayala YA, Malmierca Dr MS. Stimulus-specific adaptation and deviance detection in the inferior colliculus. Front Neural Circ. 2012;6:89. 10.3389/fncir.2012.00089 PMC3547232 23335883

[73] Ayala YA, Perez-Gonzalez D, Duque D, Nelken I, Malmierca MS. Frequency discrimination and stimulus deviance in the inferior colliculus and cochlear nucleus. Front Neural Circ. 2012;6:119. 10.3389/fncir.2012.00119 PMC3544151 23335885

[74] Pérez-González D, Hernández O, Covey E, Malmierca MS. GABA A-mediated inhibition modulates stimulus-specific adaptation in the inferior colliculus. PLOS ONE. 2012;7(3): Article e34297. 22479591 10.1371/journal.pone.0034297 PMC3315508

[75] Anderson LA, Malmierca MS. The effect of auditory cortex deactivation on stimulus-specific adaptation in the inferior colliculus of the rat. Eur J Neurosci. 2013;37(1):52–62. 23121128 10.1111/ejn.12018

[76] Duque D, Wang X, Nieto-Diego J, Krumbholz K, Malmierca MS. Neurons in the inferior colliculus of the rat show stimulus-specific adaptation for frequency, but not for intensity. Sci Rep. 2016;6:24114. 27066835 10.1038/srep24114 PMC4828641

[77] Du X, Xu H, Song R, Zhai Y, Ye H, Bao X, Huang Q, Tanigawa H, Tu Z, Chen P, et al. The multifaceted role of the inferior colliculus in sensory prediction, reward processing, and decision-making. eLife. 2025;13:RP101142. 39879260 10.7554/eLife.101142 PMC11778927

[78] Mill R, Coath M, Wennekers T, Denham SL. A neurocomputational model of stimulus-specific adaptation to oddball and Markov sequences. PLOS Comput Biol. 2011;7(8): Article e1002117. 21876661 10.1371/journal.pcbi.1002117 PMC3158038

[79] Carbajal GV, Malmierca MS. The neuronal basis of predictive coding along the auditory pathway: From the subcortical roots to cortical deviance detection. Trends Hear. 2018;22:2331216518784822. 30022729 10.1177/2331216518784822 PMC6053868

[80] Parras GG, Nieto-Diego J, Carbajal GV, Valdés-Baizabal C, Escera C, Malmierca MS. Neurons along the auditory pathway exhibit a hierarchical organization of prediction error. Nat Commun. 2017;8:2148. 29247159 10.1038/s41467-017-02038-6 PMC5732270

[81] Lao-Rodriguez AB, Przewrocki K, Perez-Gonzalez D, Alishbayli A, Yilmaz E, Malmierca MS, Englitz B. Neuronal responses to omitted tones in the auditory brain: A neuronal correlate for predictive coding. Sci Adv. 2023;9(24): Article eabq8657. 37315139 10.1126/sciadv.abq8657 PMC10266733

[82] Harpaz M, Jankowski MM, Khouri L, Nelken I. Emergence of abstract sound representations in the ascending auditory system. Prog Neurobiol. 2021;202: Article 102049. 33845166 10.1016/j.pneurobio.2021.102049

[83] Song P, Xu H, Ye H, du X, Zhai Y, Bao X, Mehmood I, Tanigawa H, Niu W, Tu Z, et al. A new function of offset response in the primate auditory cortex: Marker of temporal integration. Commun Biol. 2024;7:1350. 39424927 10.1038/s42003-024-07058-9 PMC11489726

[84] Bishop DV, Hardiman MJ, Barry JG. Is auditory discrimination mature by middle childhood? A study using time-frequency analysis of mismatch responses from 7 years to adulthood. Dev Sci. 2011;14(2):402–416. 22213909 10.1111/j.1467-7687.2010.00990.x PMC3083517

[85] Umbricht D, Schmid L, Koller R, Vollenweider FX, Hell D, Javitt DC. Ketamine-induced deficits in auditory and visual context-dependent processing in healthy volunteers: Implications for models of cognitive deficits in schizophrenia. Arch Gen Psychiatry. 2000;57(12):1139–1147. 11115327 10.1001/archpsyc.57.12.1139

[86] Du X, Song P, Gong Y, Zhai Y, Xu H, Ye H, Bao X, Huang Q, Tu Z, Chen P, et al. Protocol for behavioral and neural recording in macaques during a novelty detection task. STAR Protoc. 2024;5(3): Article 103252. 39126655 10.1016/j.xpro.2024.103252 PMC11452908

[87] Oostenveld R, Fries P, Maris E, Schoffelen JM. FieldTrip: Open source software for advanced analysis of MEG, EEG, and invasive electrophysiological data. Comput Intell Neurosci. 2011;2011: Article 156869. 21253357 10.1155/2011/156869 PMC3021840

[88] Pachitariu M, Steinmetz N, Kadir S, Carandini M. *Kilosort: Realtime spike-sorting for extracellular electrophysiology with hundreds of channels*. Cold Spring Harbor (NY): Cold Spring Harbor Laboratory; 2016.

[89] Pachitariu M, Sridhar S, Pennington J, Stringer C. Spike sorting with Kilosort4. Nat Methods. 2024;21(5):914–921. 38589517 10.1038/s41592-024-02232-7 PMC11093732

[90] Tallon-Baudry C, Bertrand O, Delpuech C, Pernier J. Stimulus specificity of phase-locked and non-phase-locked 40 Hz visual responses in human. J Neurosci. 1996;16(13):4240–4249. 8753885 10.1523/JNEUROSCI.16-13-04240.1996 PMC6579008

[91] Fisher NI. Statistical analysis of circular data. Cambridge (UK): Cambridge University Press; 1993.

[92] Ulanovsky N, Las L, Farkas D, Nelken I. Multiple time scales of adaptation in auditory cortex neurons. J Neurosci. 2004;24(46):10440–10453. 15548659 10.1523/JNEUROSCI.1905-04.2004 PMC6730303

[93] Duque D, Pérez-González D, Ayala YA, Palmer AR, Malmierca MS. Topographic distribution, frequency, and intensity dependence of stimulus-specific adaptation in the inferior colliculus of the rat. J Neurosci. 2012;32(49):17762–17774. 23223296 10.1523/JNEUROSCI.3190-12.2012 PMC6621662

[94] Ye H. Data for research.0960[DS/OL]. V1. Science Data Bank, 2025[2025-11-05]. 10.57760/sciencedb.30601. DOI:10.57760/sciencedb.30601.

