# Supplementary material for: Hierarchical Temporal Processing in the Primate Thalamocortical System: Insights from Nonlinguistic Structured Stimuli
Source: Research (Wash D C). 2025 Nov 6;8:0960. doi: 10.34133/research.0960 (PMC12676595; doi:10.34133/research.0960)
Supplement: Supplementary 1 — Figs. S1 to S7 [file research.0960.f1.zip › Supplementary Materials.docx]

**Supplementary Materials**

**Supplementary Figure 1. Anatomical location details of ECoG array and reference implantation.** (**A–B**) The exposed brain surface, including the lateral sulcus (ls) and superior temporal sulcus (sts), following the opening of the skull and dura mater in Monkey C (A) and Monkey X (B). (**C–D**) The placement of the ECoG electrodes on the brain surface in Monkey C (C) and Monkey X (D). (**E–F**) The location of the reference line (blue line) and the reference electrode (marked by black circles) in Monkey C (E) and Monkey X (F).

**Supplementary Figure 2. Evaluating temporal integration via interval insertion.** (**A**) Stimulation Design: Four distinct intervals (N = 1, 2, 4, and 8 clicks, blue) were inserted at the 4-second mark within an 8-second regular click train with a given ICI (red). Inserted ICI was 1.02 times the initial ICI. The bottom row illustrates the control sequence, which consisted of a transitional click train, composed of two 4-second click trains with the same ICIs as the above. These five patterns were presented in randomized order. (**B**) An example channel from Monkey C shows the change response to the interval-inserted click trains with different interval number (red), compared to the control condition (black). The oscillations of the responses track the frequency of the click trains. The transition to a new ICI induces a deflection of the recorded signal at 10-150 ms, which is clear for the control sequence and the 4- and 8-click insert sequences, but not when only 1 or 2 clicks are inserted in the sequence. (**C**) Average waveform showing change responses across all recording sites for Monkey C (top, initial ICI was 15.2 ms) and Monkey X (bottom, initial ICI was 14.4 ms), respectively. T = 0 ms denotes the occurrence of the first inserted interval. Horizontal gray dashed lines in (B) and (C) indicate Amplitude = 0 µV.

**Supplementary Figure 3. Phase-locking factor (PLF) to transitional click train.** (**A–B**) PLF of the segment in red background in Fig. 1B as a function of ICI for Monkey C (A) and Monkey X (B) across all tested ICI combinations. Individual light gray lines represent single recording sites, while the black lines depict the average PLF across 64 recording sites. Error bars indicate SEM. (**C**) Tonotopic distribution of PLF across all tested ICI combinations (indicated at the top of each graph) for Monkey C (top panel) and Monkey X (bottom panel). In each graph, two lines denote the lateral sulcus (upper line) and the superior temporal sulcus (bottom line), and light gray dots mark the location of ECoG electrode sites.

**Supplementary Figure 4.** **Comparative analysis of PLF between A1 and MGB responses across temporal scales.** (**A–B**) The distribution of PLF at corresponding frequencies for the first level (individual clicks) in responses to click-train-based oddball stimuli with short (A) and long (B) ICIs. The A1 responses (red) and MGB responses (blue) are compared. (**C–D**) Distribution of PLF at corresponding frequencies for the second level (click trains) in responses to click-train-based oddball stimuli with short (C) and long (D) ICIs, again comparing A1 (red) and MGB (blue). In each histogram, the vertical solid line, accompanied by a floating number, indicates the mean value.

**Supplementary Figure 5. Phase-locking factor (PLF) analysis of human EEG responses** **across temporal scales.** **(A–B)** Averaged time–frequency representations of PLF (n = 25) for two sequences (top: 18.2, 15.2, 21.9 ms; bottom: 18.2, 21.9, 15.2 ms) under individual-click level (A; the first level) and click-train level (B; the second level), aligned to the onset of the deviant train. Vertical black dashed lines mark the onset of the first standard click train in both sequences. **(A)** Left colored arrows highlight click repetition rates for the first level: 54.9 Hz (18.2 ms; gray), 65.8 Hz (15.2 ms; magenta), and 45.7 Hz (21.9 ms; blue). **(B)** Left black arrow indicates the train shifting rate (3.3 Hz) for the second level.

**Supplementary Figure6. Topographical EEG Responses to Click Trains as Deviant and Standard Stimuli.** **(A–B)** Each subplot corresponds to one EEG channel at its respective scalp location, displaying the group-averaged (n = 25) response to the same click train presented as deviant (red) or standard (black). (A) 15.2 ms ICI; (B) 21.9 ms ICI.

**Supplementary Figure 7. MRI-guided A1 and MGB neuronal recording sites.** (**A**) Three coronal brain MRI sections with highlighted regions (black boxes) in the A1 of two rhesus macaques, where neuronal recordings were conducted. Each bar represents a schematic penetration for neuronal recording, colored according to the corresponding CF. (**B**) CF distribution and partitioning of the A1, delineating specific areas: RM (rostral medial area), MM (medio-medial area), CM (caudal medial area), R (rostral area), A1 (primary auditory area), ML (mediolateral area), and CL (caudal lateral area). (**C**) Coronal brain MRI sections with blue boxes indicating the regions of the MGB in two rhesus macaques where neuronal recordings were performed. Each bar indicates a penetration for neuronal recording. (D) CF distribution of the MGB for Monkey Z (top) and Monkey M (bottom).
