# Supplementary figures and images for: Hierarchical Temporal Processing in the Primate Thalamocortical System: Insights from Nonlinguistic Structured Stimuli
Source: Research (Wash D C). 2025 Nov 6;8:0960. doi: 10.34133/research.0960 (PMC12676595; doi:10.34133/research.0960)

# Sfig1

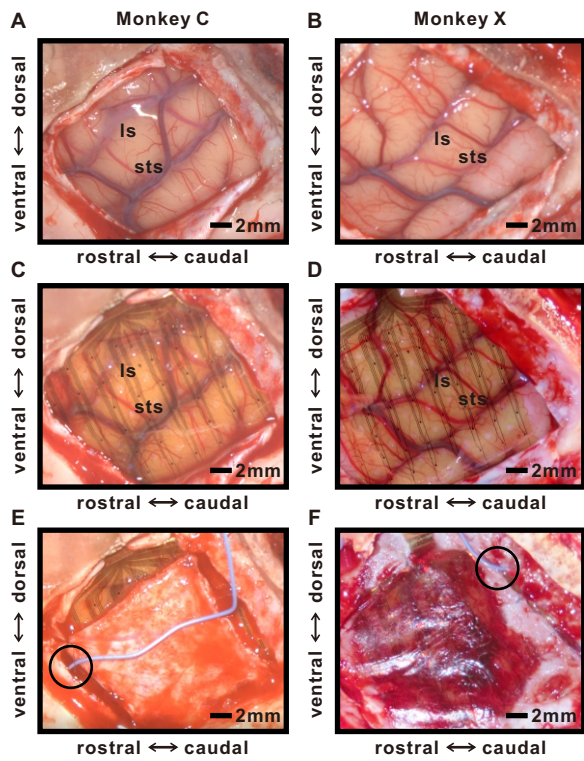

Sfig2

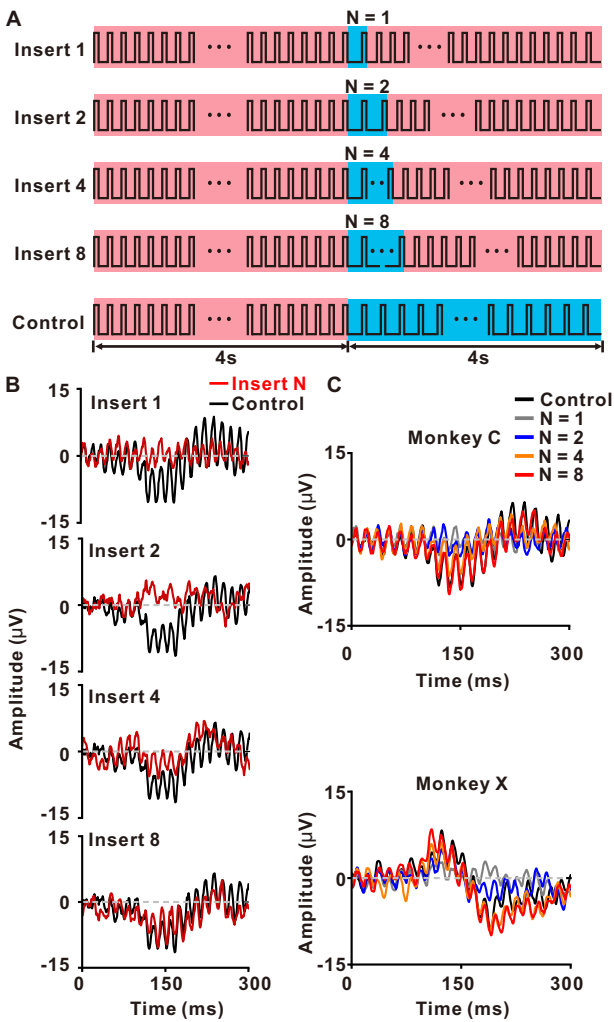

Sfig3

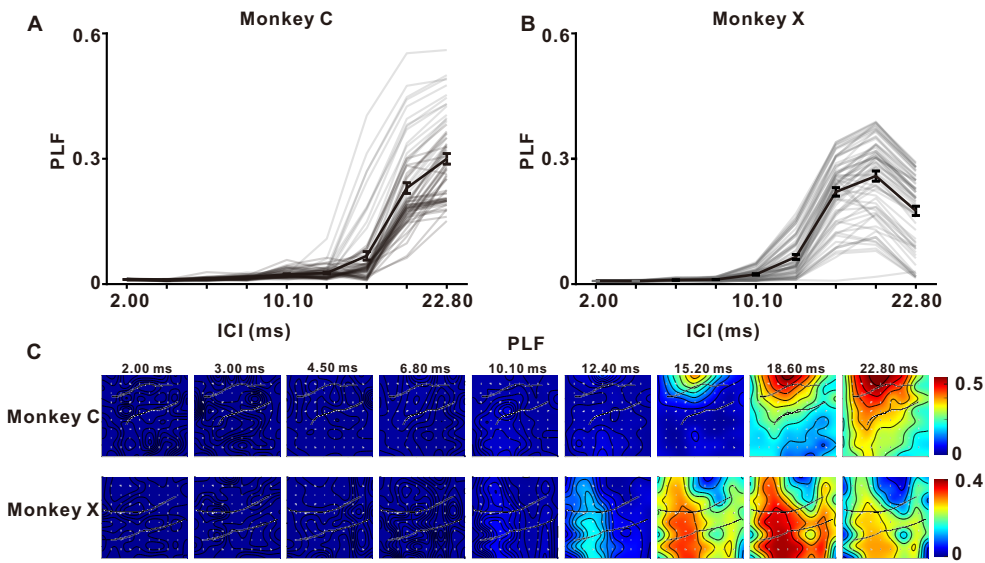

Sfig4

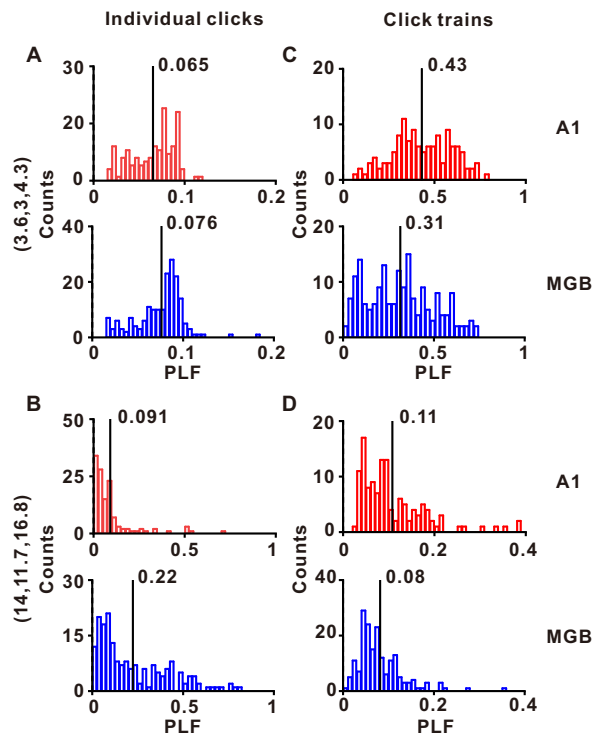

Sfig5

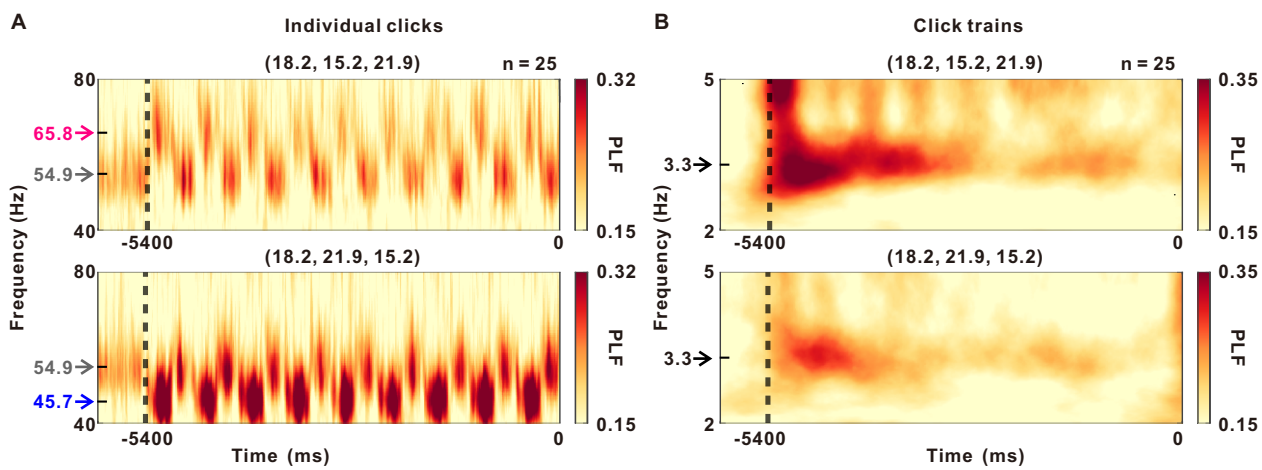

Sfig6

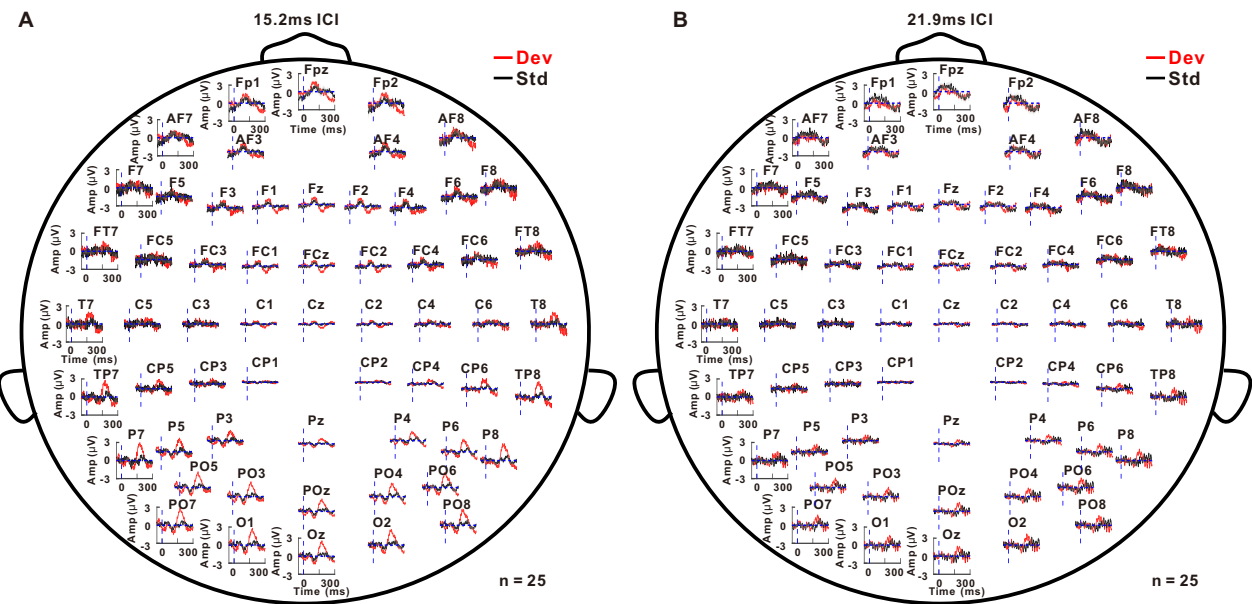

Sfig7

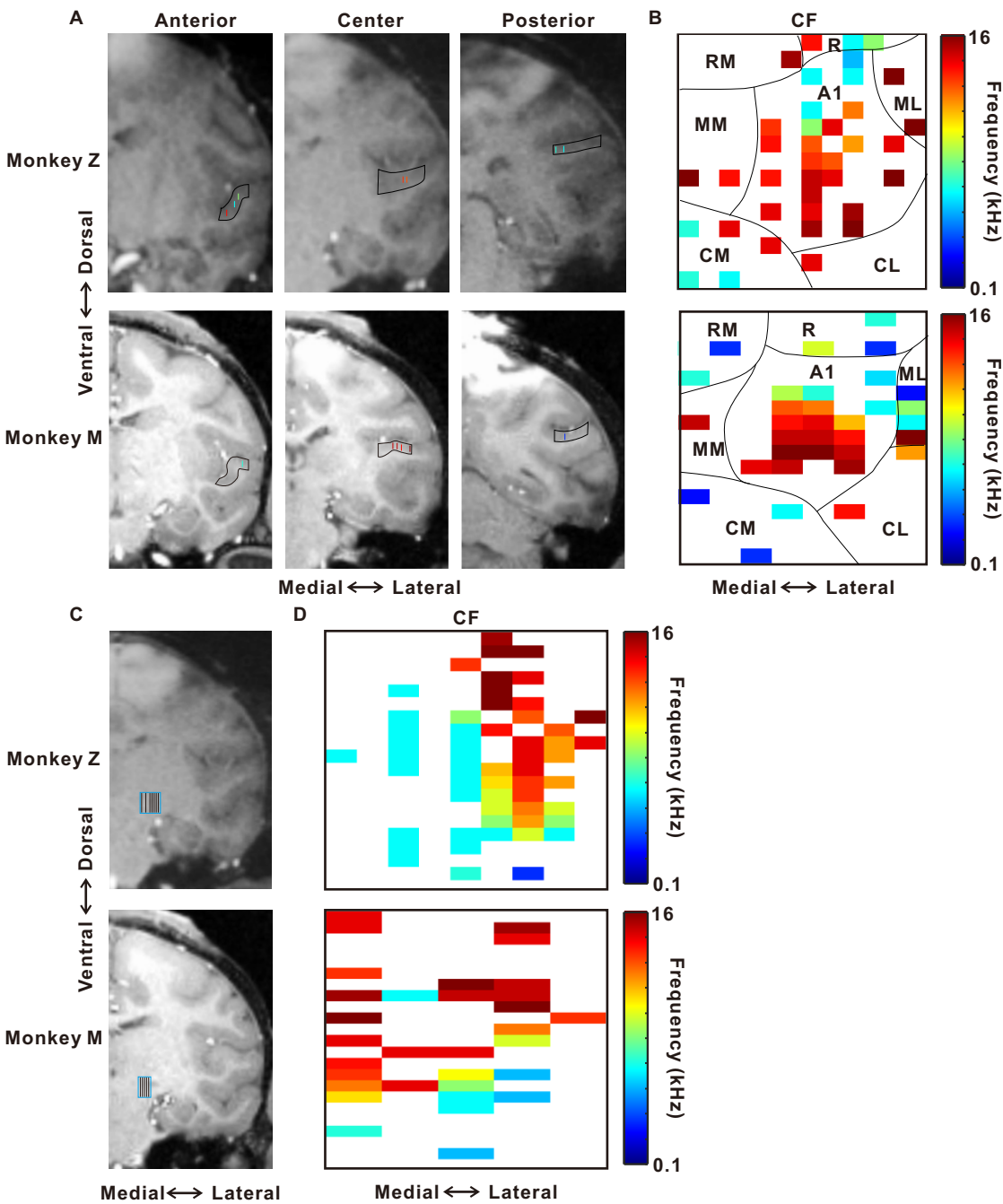

Supplement: Supplementary 1 — Figs. S1 to S7 [file research.0960.f1.zip › Figure-SM.pdf]
